# Metabolic identification of bioactive compounds of *Citrus reticulata* cultivars extracts for a novel approach to polycystic ovary syndrome

**DOI:** 10.1038/s41598-025-18116-5

**Published:** 2025-09-12

**Authors:** Shaimaa A. El Zanaty, Salwa A. Abu El Wafa, Mohammed A. Hussein, Heba A. El Gizawy, Abeer Temraz

**Affiliations:** 1 Department of Pharmacognosy, Faculty of Pharmacy, October 6 University (O6U), 6th of October City, 12585 Giza Egypt; 2 Department of Pharmacognosy and Medicinal Plants, Faculty of Pharmacy (Girls), Al-Azhar University, Cairo, 11754 Egypt; 3 Department of Biotechnology, Faculty of Applied Health Sciences Technology, October 6 University (O6U), 6th of October City, 12585 Giza Egypt

**Keywords:** Murcott, Merav, Citrus, UPLC-T-TOF-MS/MS, PCOS, COX-2, NF-kB, miR-33b-5P and miR-145, Drug discovery, Biomarkers, Diseases, Chemistry

## Abstract

Polycystic ovary syndrome; is a prevalent endocrine disorder, characterized by hormonal imbalances, inflammation, and oxidative stress. Natural remedies, including Murcott and Merav (*Citrus reticulata* varieties, Family: Rutaceae) fruits and leaves extracts, have demonstrated promising therapeutic effects in alleviating these symptoms. This study aimed to evaluate the efficacy of these extracts concerning their metabolomics in modulating hormonal levels, enhancing lipid metabolism while alleviating inflammation and oxidative stress in mice with polycystic ovary syndrome through the regulation of miR-33b-5P and miR-145 gene expression. UPLC-T-TOF-MS/MS analysis identified and quantified One hundred-seven bioactive metabolites. These metabolites were categorized into various classes according to their chemical structures, including phenolic types such as 68 flavonoids, 4 coumarins and 7 phenolic acids in addition to, 3 nitrogenous compounds, 7 terpenes and terpenoids, 6 fatty acids and their conjugates and 12 miscellaneous compounds. and various biochemical assays were performed. Blood samples were collected to measure plasma FSH, testosterone, LH, and lipid profiles. Oxidative stress biomarkers, including malondialdehyde (MDA) and superoxide dismutase (SOD), and COX-2 and NF-kB levels were assessed using ELISA kits. Micro-RNA gene expression, miR-33b-5P and miR-145, related to inflammation and oxidative stress, was analyzed using qRT-PCR. Ovarian tissues were examined histologically to assess morphological changes. These findings highlight that Murcott and Merav extracts could be a promising alternative natural treatment for PCOS, balancing hormonal levels, improving lipid profiles, and reducing inflammation and oxidative stress. Merav leaves emerged as the most promising candidate for PCOS therapy, attributed to their high levels of methylated kaempferol, a key bioactive flavonoid.

**Supplementary Information:**

The online version contains supplementary material available at 10.1038/s41598-025-18116-5.

## Introduction

Polycystic ovary syndrome (PCOS) is a prevalent endocrine condition that affects around 20% of women during their reproductive years. It is characterized by the presence of multiple ovarian cysts on ultrasound and is often accompanied by symptoms such as menstrual irregularities, excess androgen production, and challenges with fertility. It is a leading cause of female infertility and is linked to long-term metabolic and inflammatory complications. Up to 70% of affected women remain undiagnosed^1,2^. The condition is characterized by hormonal imbalances, including low follicle-stimulating hormone (FSH), elevated luteinizing hormone (LH), and high testosterone levels, which disrupt ovarian function and exacerbate symptoms such as irregular ovulation and excessive hair growth. Moreover, inflammation and oxidative stress are critical contributors to PCOS pathology, with decreased antioxidant defenses [reduced glutathione (GSH), superoxide dismutase (SOD), and catalase (CAT)] while, increased malondialdehyde (MDA), Cyclooxygenase-2 (COX-2), and Nuclear factor kappa-light-chain-enhancer of activated B cells (NF-kB) levels, contributing to disease progression^3,4^.

While pharmacological treatments for PCOS are effective in symptom management, they are frequently accompanied by undesirable side effects. This has led researchers to explore alternative therapies with better tolerability. Medicinal plants have gained attention due to their ethnopharmacological relevance and diverse phytochemical constituents, which may offer comparable benefits with reduced side effects. Herbs such as *Matricaria chamomilla* (Chamomile), *Glycyrrhiza glabra* (liquorice), *Panax ginseng*, and *Mentha spicata* (spearmint) have shown efficacy in alleviating PCOS-related issues. For instance, spearmint has been found to reduce testosterone levels and improve ovarian histology, while ginseng and liquorice help regulate blood glucose and lipid profiles, common comorbidities associated with PCOS but chamomile may help alleviate PCOS-related symptoms in ovarian tissue, promote the development of uterine follicles, and enhance LH secretion^5^. Also, *Vitex agnus-castus* (chasteberry) is well documented for its ability to regulate and maintain a healthy balance between estrogen and progesterone levels, thereby promoting menstrual regularity and reducing ovarian cyst formation. The capacity to neutralize free radicals and suppress inflammation of *Curcuma longa* (turmeric) and *Zingiber officinale* (ginger) further contribute to reducing oxidative stress, which is often elevated in PCOS patients. Additionally, plants like *Foeniculum vulgare* (fennel) and *Trigonella foenum-graecum* (fenugreek) have been noted for their estrogenic properties and ability to normalize menstrual cycles, making them valuable adjuncts in PCOS treatment^6–8^.

*C. reticulata* var. Murcott and *C. reticulata* var. Merav belong to the family Rutaceae, are both tangor hybrids and are economically valuable citrus fruits. Murcott, also known as “Honey Tangerine,” is a late-maturing variety (December–March) known for its large, juicy, seed-rich fruit with easy-to-peel skin^9^. In contrast, Merav is a smaller, seedless, sweeter mandarin with a deep orange, glossy appearance, easy to peel, and typically segmented into 7 to 14 parts^10^. *C. reticulata* was rich in phenolic compounds that are present in major concentrations^11^ while phenolic compounds have a potential activity against PCOS^12^. Kaempferol and its derivatives, a natural flavonoid, have shown promising potential in managing PCOS through their progestogenic and gene-regulatory effects. They act as a phytoprogestin that selectively modulates progesterone-related signaling pathways and downregulates PCOS-associated genes in mouse uterine tissue^13^. Additionally, kaempferol and related flavonoids have been shown in animal studies to improve ovarian structure and reduce hormonal imbalances, such as elevated LH and free testosterone levels^14^. Methylation of flavonoids such as kaempferide can show an enhanced bioavailability and metabolic stability due to the methylated form showing improved absorption compared to non-methylated counterparts^15^. Also, structural modifications like methylation may amplify anti-androgenic and antioxidant effects, which are key mechanisms in PCOS management^15,16^. This study aims to characterize the metabolite profile of Murcott and Merav fruit and leaf extracts using ultra-performance liquid chromatography combined with electrospray ionization and triple time-of-flight mass spectrometry (UPLC-ESI-T-TOF-MS/MS) and assess their impact on oxidative stress, hormonal imbalances, and ovarian function in PCOS models. By investigating their potential as natural treatments for PCOS, this research contributes to the development of alternative, plant-based therapeutic strategies.

## Materials and methods

### Plant material and extraction process

Leaves and ripe fruits rind of *C. reticulata* Blanco Cultivars; Merav and Murcott were collected from El-Batoul Farm for Citrus fruits, Nubariah City, El-Beheira Governorate, Egypt in June 2023 for the leaves and in February 2024 for the ripe rind. Professor Dr. Wafaa Amer, who specializes in Taxonomy at Cairo University in Egypt, confirmed their identities. In the herbarium of the Department of Pharmacognosy and Medicinal Plants, Faculty of Pharmacy (Girls), Al-Azhar University, four samples have been placed with vouchers CRL23 for Merav leaves in 2023, CML23 for Murcott leaves in 2023, CRF24 for Merav fruit rind in 2024 and CMF24 for Murcott fruit rind in 2024.

Leaves and ripe fruits rind of *C. reticulata* Blanco Cultivars; Merav and Murcott (2 kg, each) were powdered and extracted separately in a Soxhlet (Alderich^®^ soxhlet, Darmstadt, Germany) with methanol (3 × 5 L, 70%) under reflux (65 °C). After filtration, the methanol extracts were concentrated at 45 °C under vacuum using a BÜCHI Rotavapor^®^ system to obtain a brown crude extract of 160, 200, 190 and 250 g for Murcott fruit (MTF), Murcott leaves (MTL), Merav fruit (MVF) and Merav leaves (MVL), respectively. The obtained dried extracts were used for biological and chemical investigations^17^. All methods were carried out in accordance with relevant guidelines and regulations.

The dose of 500 mg/kg b.w. for Murcott and Merav fruit and leaf extracts was selected based on dose ranges commonly used in phytopharmacological investigations evaluating the anti-inflammatory, antioxidant, and hormone-modulating activities of citrus-derived phenolics. According to Zhang et al. (2022)^14^ and Yuan et al. (2025)^12^plant extract doses between 200 and 600 mg/kg b.w. have shown reproducible therapeutic benefits in PCOS animal models.

### UPLC-ESI-T-TOF-MS/MS analysis

UPLC-ESI-T-TOF-MS/MS was employed to analyze active fractions (MTL, MVL, MTF, and MVF) at the Proteomics and Metabolomics Lab, Children’s Cancer Hospital Egypt 57,357. The analysis utilized a Waters UPLC system with an X Select HSS T3 column (2.5 μm, 2.1 × 150 mm) and an in-line filter (0.5 μm pore size, 3.0 mm diameter) to prevent particulate contamination. Sample preparation involved dissolving 50 mg of each extract in a 50:25:25 mixture of deionized water, methanol, and acetonitrile, followed by vortexing, sonication, and centrifugation. A 10 µL aliquot of the prepared solution (2.5 µg/µL) was injected for analysis. Using UPLC-ESI-T-TOF-MS/MS, phytochemical profiling was achieved under both negative and positive ionization conditions. A gradient elution was performed with solvent A (5 mM ammonium formate buffer, pH 8, with 1% methanol) and solvent B (100% acetonitrile) at a flow rate of 0.3 mL/min. Data collection and analysis were conducted using Peak View 2.2 and MS-DIAL 4.6. Tentative compound identification was achieved by comparing retention times, molecular masses, and fragmentation patterns against databases including Respect (positive and negative), the Human Metabolome Database (HMDB), and NIST libraries, as well as published literature^18–20^.

### Experimental design

Forty-two albino mice, weighing 35 ± 2.5 g each, were purchased from the Cancer Institute at Cairo University. Animal studies were performed in accordance with ARRIVE guidelines with ethical approval granted by the Research Ethics Committee of the Faculty of Pharmacy for Girls, Al-Azhar University, Egypt (Ethics Approval No. 468).

The mice were housed in polypropylene cages with industry-standard humidity levels and a natural light-dark cycle. They had access to water and normal pellets, which were supplied by the Cantacuzino Institute in Bucharest, Romania. Dyets Inc. (Bethlehem, PA, USA) provided the regular diet. The high-fat diet (HFD) consisted of 200 g of fat per kg and 1% (w/w) cholesterol, as previously described by Shahid et al.^21^ and Kafali et al.^22^ to induce PCOS-like metabolic disturbances in rodents. Seven groups were formed by randomly dividing the mice, with six mice in each group, as illustrated in (Table 1). At the end of the experiment, cervical dislocation was used for euthanizing animals. It involved the rapid dislocation of the cervical vertebrae from the skull. The procedure is performed swiftly to ensure immediate unconsciousness and death.

**Table 1:** Treated animal grouping and description.

Groups	Group name	Treatment description
I	Normal control	Mice were fed a normal diet and 3 mL of distilled water orally for 8 weeks
II	PCOS-induced group [HFD + Letrozole (90 µg /kg.b.w.)]	Received HFD for 8 weeks^21^ and given letrozole by gavage (90 µg/kg.b.w) daily for the last 3 weeks^22^. The dosage of letrozole (90 µg/kg b.w.) used to induce PCOS in mice is based on the protocol established by Kafali et al.,^22^which reliably reproduces PCOS-like ovarian cysts and hormonal disturbances in rodents.
III	PCOS + MTF (500 mg/kg.b.w)	Given HFD for 8 weeks + Letrozole by gavage (90 µg/ kg.b.w) + MTF (500 mg/ kg. B.W.) suspended in distilled water daily along with letrozole for the last 3 weeks.
IV	PCOS + MTL (500 mg/kg.b.w)	Given HFD for 8 weeks + Letrozole by gavage (90 µg/ kg.b.w) + MTL (500 mg/ kg. B.W.) suspended in distilled water daily along with letrozole for the last 3 weeks.
V	PCOS + MVF (500 mg/kg.b.w)	Given HFD for 8 weeks + Letrozole by gavage (90 µg/ kg.b.w) + MVF (500 mg/ kg. B.W.) suspended in distilled water daily along with letrozole for the last 3 weeks.
VI	PCOS + MVL (500 mg/kg.b.w)	Given HFD for 8 weeks + Letrozole by gavage (90 µg/ kg.b.w) + MVL (500 mg/ kg. B.W.) suspended in distilled water daily along with letrozole for the last 3 weeks.
VII	PCOS + Metformin (300 mg/kg.b.w)	Given HFD for 8 weeks + Letrozole by gavage (90 µg/ kg.b.w) + Metformin (300 mg/kg b.w) suspended in distilled water daily along with letrozole for the last 3 weeks. The selected dose of metformin (500 mg/kg b.w.) was based on prior in vivo studies demonstrating therapeutic efficacy in improving hormonal, metabolic, and ovarian dysfunctions in PCOS animal models without inducing systemic toxicity. A comparable dose was used by Di Pietro et al. (2015)^23^who reported significant modulation of ovarian angiogenesis and follicular development using metformin in a letrozole-induced PCOS rat model.

HFD: high fat-diet, PCOS: polycystic ovary syndrome induced group, MTF: Murcott fruit, MTL: Murcott leaves, MVF: Merav fruit, MVL: Merav leaves, B.W: body weight.

Body weights were recorded every two weeks. At the end of the experiment, the mice were anesthetized with 50 mg of thiopental sodium and then sacrificed for blood and tissue collection. Blood samples were collected in fluoride tubes and centrifuged^24^. Plasma levels of FSH, testosterone, and LH were measured using ELISA kits from Abbexa (UK), My Biosciences (USA), and Abcam (USA). Plasma COX-2 levels were determined using an ELISA kit (ab210574, Abcam, USA). Immediately after isolation, the ovaries were washed with saline and divided into three sections. The first Sect. (100 mg) was homogenized in 1 ml of phosphate-buffered saline (pH 7.4) for 15 min. The homogenate was split into two parts, with the first part being centrifuged at 1000 rpm and filtered. The obtained supernatant was used for the estimation of GSH, SOD, CAT and MDA. A chloroform-methanol mixture (2:1, 1 ml) was used to extract the second portion of the homogenate (0.2 ml), and the extract was then concentrated using a nitrogen stream. This extract was used for the estimation of ovarian NF-kβ and MDA using ELISA kits (ab176648, Abcam, USA) and (MBS741034, My Biosciences, USA), respectively^25–27^.

### Quantitative Real-Time PCR

Using Sepasol-RNA1Super (Nakarai Tesque), total RNA was extracted from a portion of the ovarian tissue designated for gene expression analysis as per the manufacturer’s protocol. Real-time quantitative PCR (RT-PCR) was used to assess miR-33b-5P and miR-145 gene expression in Sects. (10–15 g) of the recovered RNA. The PCR reaction mixture comprised PCR buffer, 1.5 mM MgCl_2_, 0.2 mM of each dNTP, and 0.4 µM of the appropriate primers (Table 2), with assays conducted in a 50 µl single-plex mixture. The PCR conditions included 40 cycles of 95 °C for 15 s and 60 °C for 1 min, with pre-incubation steps at 50 °C for 2 min and 95 °C for 10 min. GAPDH mRNA was used for internal control normalization^28^.

**Table 2:** Primer’s sequence used in real-time PCR.

Gene	Primer sequence
miR-33b-5P	F:5′-CTCAACTGGTGTCGTGGAGTC GGCAATTCAGTTGAGTGCAATGC-3′ R:5′-ACACTCCAGCTGGGGTGCATTGTAGTTGCAT-3′
miR-145	F: 5′- ACACTCCAGCTGGGCAGGTCAAAAGGGTCC-3 R: 5′- TGTGAGGTCGACCCGTCCAGTTTTCCCAGG-3
GAPDH	F: 5’-TCGGAGTCAACGGATTTGGT-3’ R: 5’-TTCCCGTTCTCAGCCTTGAC-3’

### Histological assessment

After fixation in 10% neutral formalin, the third part of the ovarian tissue was dehydrated in graded alcohol, embedded in paraffin, and sectioned. The slices were stained with Hematoxylin and Eosin (H&E), mounted on slides, and analyzed under a light microscope according to the Bancroft and Steven method^29^.

### Statistical evaluation

Gene expression was measured quantitatively by PCR in triplicate, and spectrophotometric and ELISA analyses were conducted in six separate replicates. The results were presented as mean ± standard deviation (SD). Statistical comparisons were made using SPSS version 20, applying one-way ANOVA followed by the Bonferroni post hoc test for multiple comparisons. A *p*-value of < 0.05 was considered statistically significant^30^.

## Results and discussion

### UPLC-T-TOF-MS/MS analysis of Murcott and Merav leaves and fruits

**Fig. 1:**
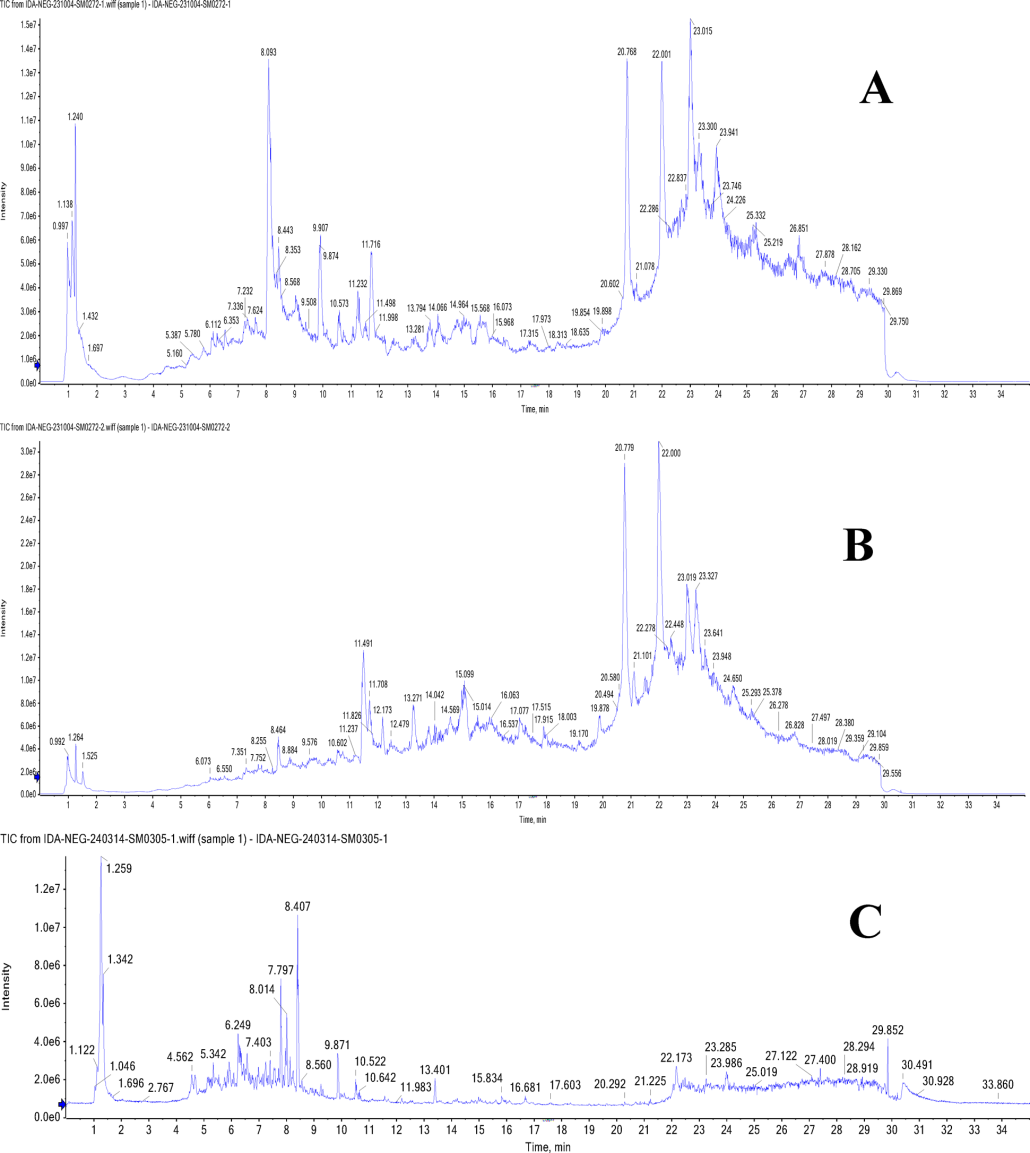

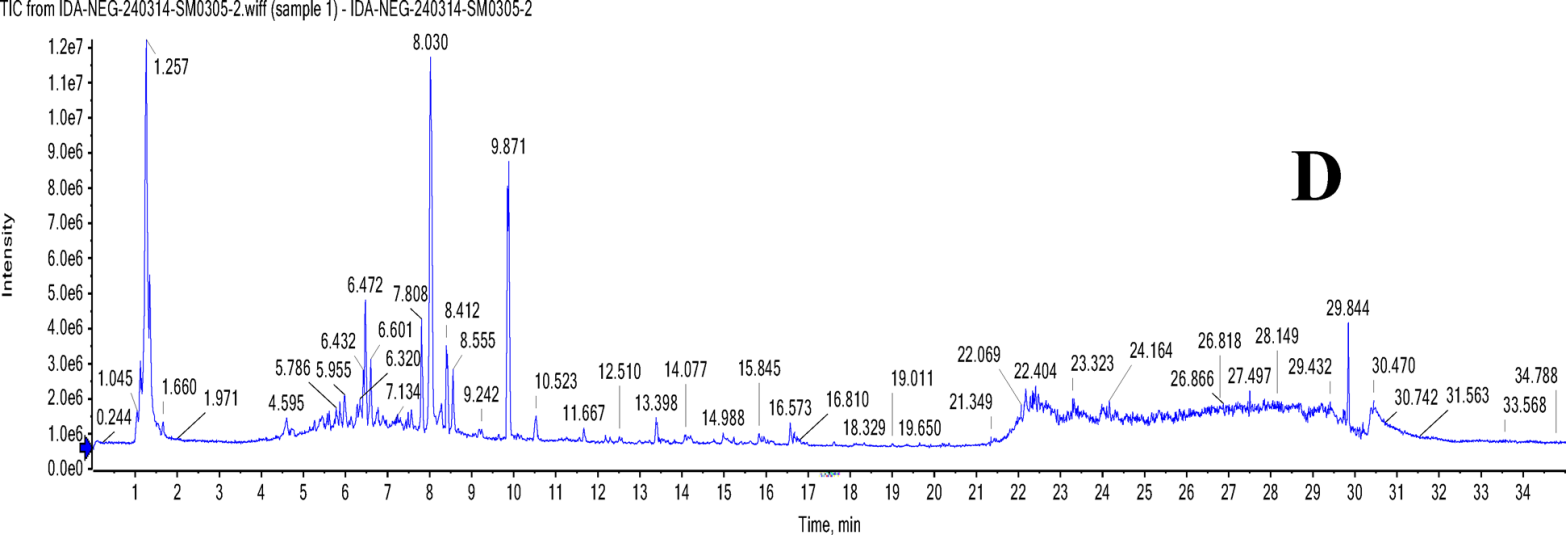
TIC of MTL (**A**), MVL (**B**), MTF (**C**) and MVF (**D**) using UPLC-ESI-T-TOF-MS/MS in negative ionization mode.

**Fig. 2:**
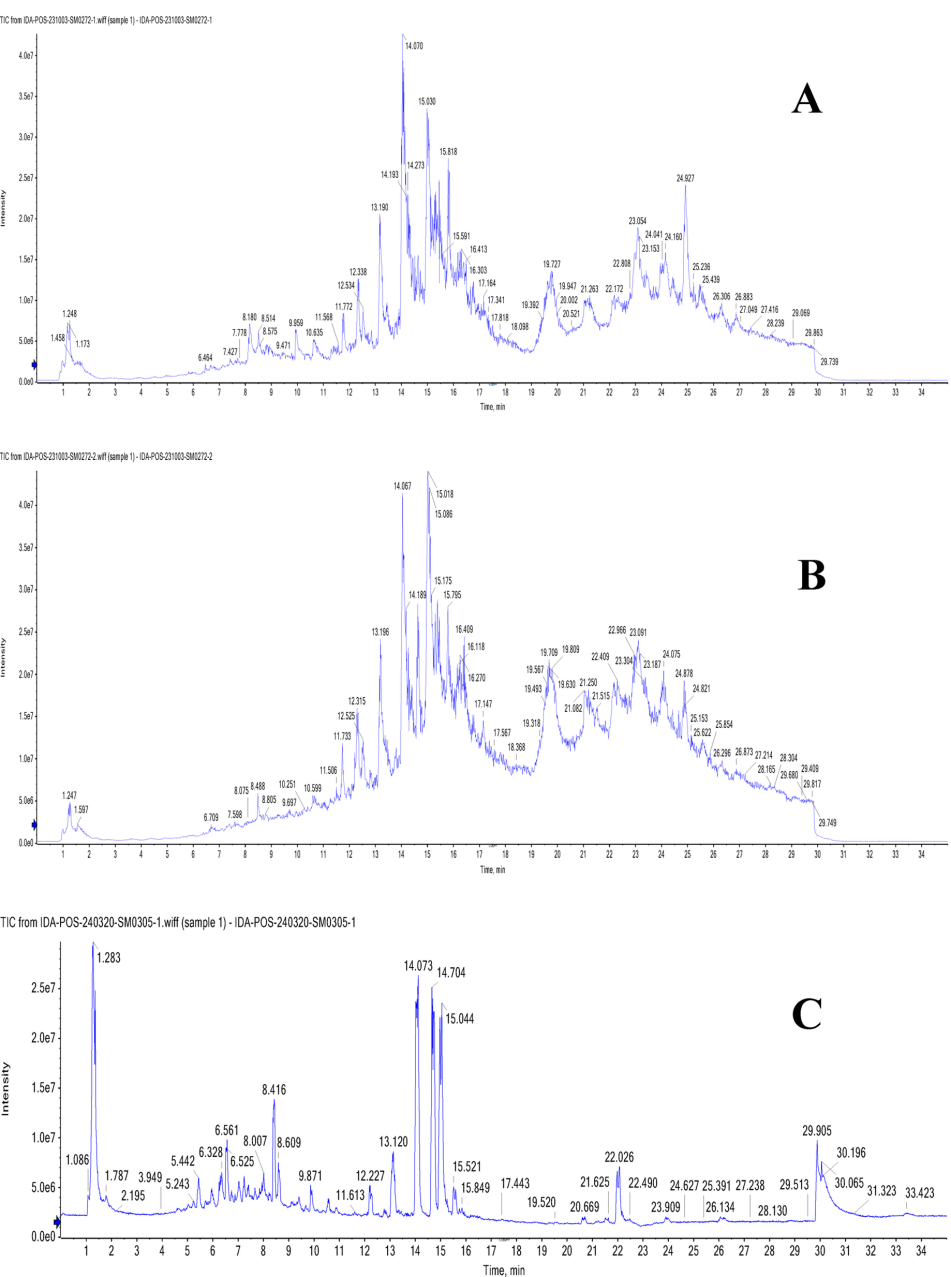

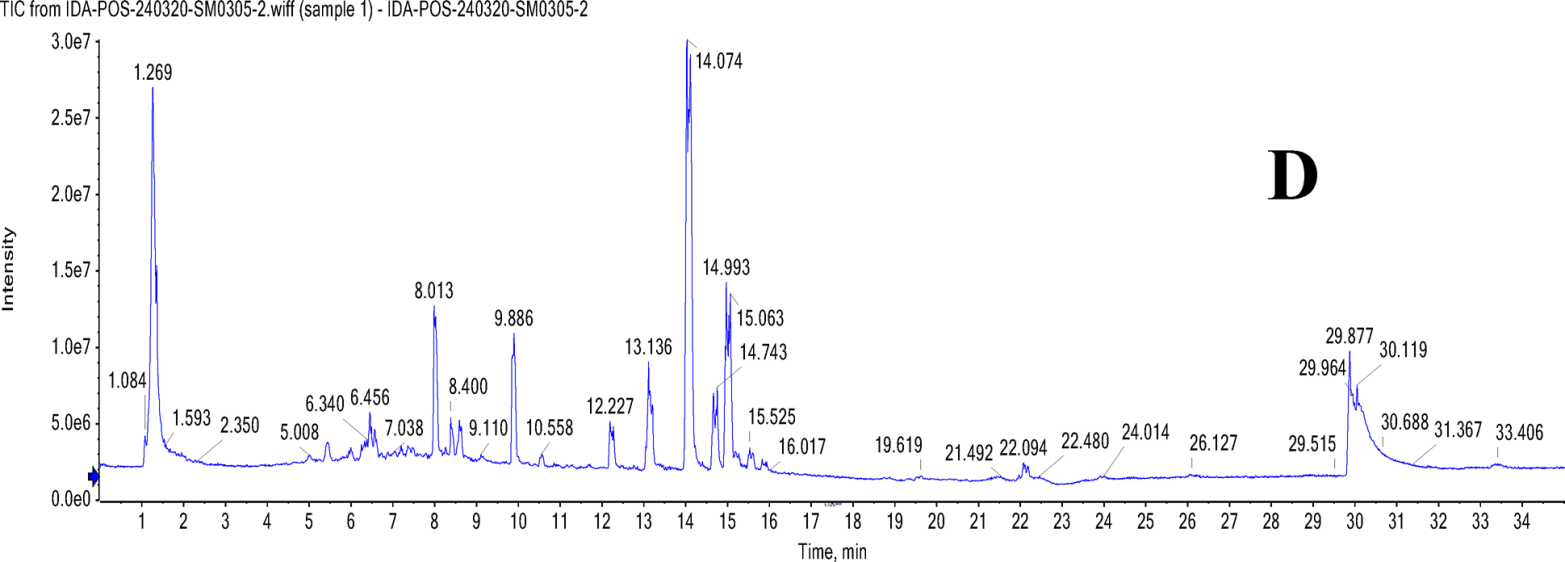
TIC of MTL (**A**), MVL (**B**), MTF (**C**) and MVF (**D**) using UPLC-ESI-T-TOF-MS/MS in positive ionization mode.

The leaves and fruits rind of *C. reticulata* var. Murcott and Merav showed a wide variety of polyphenolic metabolites, according to the total ion chromatograms (TIC) in negative and positive modes (Figs. 1 and 2**).** For the identified metabolites, Table 3 presents the retention times (R_t_), observed molecular masses *m/z*, molecular formulas (MFs), selected daughter fragments and error (ppm) values. The elution order was used to assign the peak numbers. In total, one hundred-seven metabolites were detected of diverse chemical classes in both parts of Murcott and Merav varieties extracts. The identification of these metabolites is predicated on the confirmation of the aforementioned corresponding parameters in conjunction with the extracted ion chromatograms (XIC), tandem mass spectral selective fragments in MS^1^ and/or MS^2^ two-stage fragmentation spectra, and their complete correspondence with the information reported for each identified metabolite and the corresponding convenient library database (MoNA; Mass Bank of North America). These metabolites were categorized into various classes according to their chemical structures, including phenolic types such as 68 flavonoids, 4 coumarins and 7 phenolic acids. In addition to, 3 nitrogenous compounds, 7 terpenes and terpenoids, 6 fatty acids and their conjugates and 12 miscellaneous compounds. Numerous flavonoid types, including flavanones, flavones, isoflavones, flavonols, anthocyanidins, aurones, chalcones, and their related glycosides, were found in *C. reticulata*. Sixteen flavonoid aglycone and/or their glycosides were present in all extracts of MTL, MTF, MVL and MVF represented in Table 3 peaks ID. **(1/2/5/6/7/8/14/15/24/28/34/37/54/56/59/64)**. In addition, there are twenty-three flavonoid aglycone and/or glycosides found in the fruits rind of Murcott and/ or Merav while absent in their leaves **(3/4/16/20/22/25/26/27/29/32/36/38/39/43/44/46/51/58/60/62/63/65/67)**. Twelve were detected only in MTL and/ or MVL **(9/17/18/23/30/35/40/45/47/49/52/57)**.

#### Flavanones

As shown in Table 3, a total of nine flavanones were identified. Six of these were found across all four extracts, corresponding to peaks 1, 2, 5, 6, 7, and 8. Peaks 3 and 4 were detected only in the MTF extract, while the ninth peak appeared exclusively in the MTL extract. Naringenin, a flavanone predominantly found in Citrus fruits, was assigned to peak (7). Its MS² spectrum displayed a deprotonated molecular ion [M–H]⁻ at *m/z* 271.0605, with two characteristic daughter ions at *m/z* 151.0036 [M − H−C₈H₈O]⁻ and *m/z* 119.0498 [M − H−C₇H₄O₄]⁻, which were created by the Retro Diels-Alder reaction on the chemical bonds 1 and 3^31^. The second flavanone common to all four extracts was peak (1), which displayed a deprotonated ion [M–H]⁻ at *m/z* 433.1185. The MS² spectrum showed a major fragment ion at *m/z* 271.0610 [M − H−162]⁻, indicating the loss of a hexose moiety and suggesting naringenin as the aglycone. Protonated ions [M + H]⁺ at *m/z* 435.1262 and aglycone ion at *m/z* 273.0559 [M + H − 162]⁺ were also detected. The fragmentation pattern matched well with the database for naringenin 7*-O-*hexoside^32^. Similarly, peak (6), present in all four extracts, showed a protonated molecular ion [M + H]⁺ at *m/z* 595.2019 and a deprotonated ion [M–H]⁻ at *m/z* 593.1902. A characteristic aglycone ion appeared at *m/z* 287.0906 [M + H − 309]⁺, suggesting the compound is isosakuranetin 7*-O-*neohesperidoside, an *O*-methylated flavonoid and a 4’-methoxy derivative of naringenin^33^. Peak (8) showed a deprotonated molecular ion [M–H]⁻ at *m/z* 301.0720 and a protonated ion [M + H]⁺ at *m/z* 303.0844. Its MS² spectrum exhibited a key fragment at *m/z* 286.0478 [M–H–CH₃]⁻ and additional fragments matching the reported fragmentation of hesperetin, the 4’-methoxy derivative of eriodictyol^34^. Peak (5) displayed [M–H]⁻ at *m/z* 609.1833 and [M + H]⁺ at *m/z* 611.2517. The MS² spectrum revealed a fragment at *m/z* 301.0713 [M–H–rutinoside]⁻, characteristic of the hesperetin aglycone. Based on the structure, it was identified as hesperidin, a disaccharide derivative of hesperetin linked via a glycosidic bond to a rhamnosyl-glucosyl moiety^35^. Peak (4), present only in MTF showed a deprotonated molecular ion [M–H]⁻ at *m/z* 609.1738. The MS² spectrum exhibited a fragment at *m/z* 301.0713 [M–H − 308]⁻, corresponding to the loss of a neohesperidoside moiety (308 Da), characteristic of the hesperetin aglycone. Thus, it was tentatively identified as hesperetin 7*-O-*neohesperidoside^35^. Peak (2), present in all four extracts, showed a deprotonated molecular ion [M–H]⁻ at *m/z* 595.1640. The MS² spectrum revealed product ions at *m/z* 449.1302 [M–H–146]⁻ and 287.0571 [M–H–308]⁻, indicating losses of rhamnose and neohesperidoside moieties, respectively. It was tentatively identified as eriodictyol 7*-O-*neohesperidoside (neoeriocitrin)^31^. Finally, flavanomarein, eluted exclusively from MTF (peak 3), exhibited a molecular ion [M–H]⁻ at *m/z* 449.1027. The MS² spectrum showed fragment ions at *m/z* 287.0549 [M–H–glucose]⁻ and 269.0427 [M–H–glucose–H₂O]⁻, along with cleavage fragments at *m/z* 151.01132 and 135.0497 due to cleavage of the C-ring, allowing for tentative identification as isookanin 7*-O-*hexoside^36^.

#### Chalcones

Three chalcones were identified (Table 3), all exhibiting fragmentation patterns similar to those of flavanones. Marein (peak 66) was detected exclusively in Merav extracts and displayed a molecular ion [M–H]⁻ at *m/z* 449.0889 and a fragment at *m/z* 287.2334 [M–H–162]⁻, corresponding to the loss of a glucose moiety. Based on these data, it was tentatively identified as okanin 4’*-O-*hexoside (marein)^36^. Neohesperidin dihydrochalcone (peak 67) was detected only in MTF, its MS spectrum showed a molecular ion [M–H]⁻ at *m/z* 611.1354 along with characteristic fragment ions resulting from the sequential loss of rhamnose and glucose units, as well as multiple (CH₂O) losses typical of C-glycosides^37^. Additionally, phlorizin (peak 68) was identified exclusively in Murcott extracts. It exhibited a protonated molecular ion [M + H]⁺ at *m/z* 437.1937 and a deprotonated ion [M–H]⁻ at *m/z* 435.2028, releasing a major fragment at *m/z* 273.1481 [M–H–162]⁻, corresponding to the loss of a hexosyl moiety, thus confirming its identity as phlorizin^38^.

#### Flavones

The main fragmentation pathway for flavone aglycones is the retro-Diels-Alder (RDA) reaction, often accompanied by the loss of small neutral molecules such as H₂O, CO, CO₂, and C₃O₂. In this study, fifteen flavones (peaks 10–24) were identified, mainly corresponding to luteolin and apigenin derivatives. Among the luteolin derivatives, peak 24 was identified as luteolin (5,7,3’,4’-tetrahydroxyflavone), showing strong signals at [M + H]^+^ *m/z* 287.0881 and [M-H]^−^ *m/z* 285.0768, with fragmentation patterns consistent with known standards^39^. Peak 10 was assigned to luteolin 7,3’-di*-O-*hexoside ([M-H]^−^ at *m/z* 609.0918), with a characteristic fragment ion at *m/z* 283.0968 [M-3 H-2 × 162]^-^ identified through the loss of two hexosyl units^40^. Peaks 11 and 12 were identified as luteolin 8*-C-*glucoside (orientin) and luteolin 6*-C-*glucoside (isoorientin), respectively, both displaying a [M-H]^−^ ion at *m/z* 447.0936 and 447.0912, respectively. And distinguished by characteristic sugar cleavages ([M-H-90]^−^ and [M-H-120]^−^) in their MS² spectra^41^ (Table 3). Peak 22 was confirmed as luteolin 4’*-O-*hexoside ([M + H]^+^ at *m/z* 449.1597), based on the observed loss of a glucose moiety^42^.

Regarding apigenin derivatives, peak 14 was identified as apigenin 8*-C-*glucoside (vitexin) with [M + H]^+^ at *m/z* 433.1492, characterized by typical C-type hexoside fragment losses^43^. Peak 15 corresponded to apigenin 7*-O-*neohesperidoside (rhoifolin) ([M-H]^−^ at *m/z* 577.1533), fragmenting to yield apigenin ([*m/z* 269.0453 M-H-146-162]^-^)^44^. Peak 18 was identified as apigenin 7*-O-*hexoside ([M-H]^−^ at *m/z* 431.0963), showing the loss of a hexosyl moiety (*m/z* 268.-390 [M-2 H-162]^-^)^45^. Peak 20 was assigned to acacetin 7*-O-*neohesperidoside ([M-H]^−^ at *m/z* 591.1814) based on the loss of a neohesperidoside group^45^while peak 23 was confirmed to be apigenin aglycone ([M-H]^−^ at *m/z* 269.0452) by characteristic C-ring cleavages (*m/z* 117.0344 and 151.0038)^46^. Peak 19 was identified as acacetin (the 4’-methyl ether derivative of apigenin) with a deprotonated molecular ion at *m/z* 283.0620^47^, and peak 21 corresponded to acacetin 7*-O-*rutinoside (linarin) ([M-H]^−^ at *m/z* 591.3115), indicated by the loss of a rutinoside moiety^20^. Finally, peak 17 was identified as baicalein 7*-O-*glucuronide ([M-H]^−^ at *m/z* 445.1099), based on sequential neutral losses of CO, CO₂, H₂O, and a glucuronide moiety^39^.

#### Isoflavones

Six isoflavones (peaks 25–30) were identified as either aglycones or glycosides. Peak 25, detected only in MVF, exhibited a protonated molecular ion at *m/z* 255.1450. Its Ms^2^ spectrum exhibited product ions at *m/z* 236.9890 [M + H-H_2_O]^+^, 165.9184 [M + H-H_2_O-CO-CO_2_]^+^and 122.9401 [M + H-3CO_2_]^+^ all these data matched with daidzein^48^. Peak 28 was found in all samples. It demonstrated a deprotonated molecular ion at *m/z* 415.1227 [M − H]^−^. The MS² spectrum revealed a major fragment ion at *m/z* 371.1811 [M − H−CO_2_]^−^, aligning with puerarin (daidzein 8*-C-*hexoside)^48^. Peaks 27 and 29 were observed in both MTF and MVF. Peak 27 showed a molecular ion at *m/z* 271.1186 [M + H]^+^, with fragment ions resulting from losses of water (*m/z* 252.9485 [M + H − H_2_O]^+^) and carbon monoxide (*m/z* 242.9196), supporting its identification as genistein^49^. Peak 26, present only in MTF, exhibited a precursor ion at *m/z* 431.1562. Fragmentation produced ions at *m/z* 269.1088 (loss of glucosyl group, − 162 Da) and 226.9483, with further loss yielding a fragment at *m/z* 254.0554 (loss of CH₃ and CO). This matched the reported pattern for ononin^50^. Peak 29 was assigned to 5-hydroxy ononin (sissotrin), showing a protonated ion at *m/z* 447.1677 [M + H]^+^. Its MS² spectrum displayed the aglycone ion at *m/z* 285.1139 [M + H − 162]^+^, indicating the loss of a glucosyl group^50^. Finally, Peak 30, detected only in MVL, showed a deprotonated ion at *m/z* 267.1938 (C₁₆H₁₂O₄). Fragmentation led to four product ions at *m/z* 252.0462 (loss of CH₃, − 15 Da), 224.0521 (loss of CH₃+CO, − 43 Da), 211.0458 (loss of 2CO, − 56 Da), and 195.0514 (loss of CO + CO₂, − 72 Da), identifying it as formononetin^51^.

#### Flavonols

In the two plants studied, a total of twenty-six flavonols were detected, including eight kaempferol derivatives, nine quercetin derivatives, three isorhamnetin derivatives, two syringetin derivatives, two myricetin derivatives, and individual detections of taxifolin and gossypin. Taxifolin was identified exclusively in the MTF sample (peak 32) with a molecular ion at *m/z* 305.1297 [M + H]^+^ and a characteristic fragment at *m/z* 287.1295 [M + H–18]^+^, consistent with loss of H₂O^52^. Two peaks were attributed to myricetin derivatives; peak 31, identified as myricetin aglycone, was present in MTL, MVL, and MVF), showing [M + H]^+^ at *m/z* 319.0969 and [M–H]^–^ at *m/z* 317.0550^53^. Also, peak 47 was identified as myricetin 3*-O-*rhamnoside (myricitrin), observed only in MTL, with [M–H]^–^ at *m/z* 463.1173^54^. Kaempferol derivatives were widely detected. Peak 33, found in MTL and MTF, showed [M–H]^–^ at *m/z* 461.1724 and a fragment at *m/z* 285.09616, indicating loss of a glucuronide moiety, tentatively identified as kaempferol 3*-O-*glucuronide^54^. Peak 34 was attributed to kaempferol 7*-O-*neohesperidoside^54^ with [M–H]^–^ at *m/z* 593.1493 and a fragment at *m/z* 285.0437. Peak 38, seen in MTF and MVF, was identified as kaempferol 3*-O-*robinoside-7*-O-*rhamnoside, with [M + H]^+^ at *m/z* 741.2368 and fragments from rhamnose and robinose losses^55^. Peak 42, with [M–H]^–^ at *m/z* 431.1905 and a loss of rhamnose to yield *m/z* 285.1485, was assigned as kaempferol 3*-O-*α-L-rhamnoside^56^. Peaks 44 to 46 indicated various kaempferol glycosides: peak 44 (kaempferol 3*-O-*rutinoside)^57^ with [M + H]^+^ at *m/z* 595.1741 and fragment at *m/z* 287.07844; peak 45 (kaempferol 3*-O-*(6*-p-*coumaroyl)-hexoside)^57^ with [M–H]^–^ at *m/z* 593.1507 and fragment at *m/z* 285.0403; and peak 46 (kaempferol 3*-O-*hexoside)^58^ with [M + H]^+^ at *m/z* 449.1830 and fragment at *m/z* 287.1305. Peak 54 present in the four plants, was assigned as kaempferide (kaempferol 4’*-O-*methyl ether)^59^with [M–H]^–^ at *m/z* 299.0562 and a product ion at *m/z* 284.0330. Syringetin derivatives were represented by peaks 48 and 49, both with [M–H]^–^ at *m/z* 507.1148 and 507.1167, respectively, and aglycone ions at *m/z* 345.0618 and 344.0563 due to loss of hexoside moiety. These were identified as syringetin 3*-O-*galactoside and syringetin 3*-O-*hexoside, respectively^60^. Quercetin derivatives included peaks 35, 39, 40, 41, 43, 51, 52, 53, and 56. Peak 53 (quercetin aglycone) was present in MTL and MTF, showing [M–H]^–^ at *m/z* 301.0736 and [M + H]^+^ at *m/z* 303.1436 with typical RDA fragments^31^. Peak 40 was identified as quercetin 3*-O-*α-L-rhamnoside (quercitrin)^61^ with [M–H]^–^ at *m/z* 447.1960 and a fragment at *m/z* 301.1678. Peak 43, identified as quercetin 3*-O-*rutinoside (rutin)^62^showed [M–H]^–^ at *m/z* 611.1714 and a fragment at *m/z* 303.0598. Peaks 51 and 52 were assigned to isoquercitrin and quercetin 4’*-O-*hexoside based on MS^2^ fragments at *m/z* 303.0920 and *m/z* 301.0728, respectively^63^. Peak 35, seen only in MTL, showed [M–H]^–^ at *m/z* 433.1754 and a fragment at *m/z* 301.1076, identified as quercetin 3*-O-*xyloside^64^. Isorhamnetin derivatives were represented by peaks 37, 50, and 55. Peak 37 was identified as isorhamnetin 3*-O-*rutinoside with [M–H]^–^ at *m/z* 623.1601 and fragment at *m/z* 315.1159. Peak 50, with [M–H]^–^ at *m/z* 477.1062 and a fragment at *m/z* 315.0541, was assigned as isorhamnetin 3*-O-*hexoside^65^. Finally, peak 55, prominent in MTL, was identified as isorhamnetin aglycone with [M–H]^–^ at *m/z* 315.0877 and [M + H]^+^ at *m/z* 317.0682, confirming it as 5,7,4’-trihydroxy-3’-methoxy flavonol^65^.

#### Anthocyanin

A total of seven anthocyanin-type flavonoids were identified, with only peaks 59 and 64 being common to all four extracts, while peaks 62, 63, and 65 were exclusive to MTF, peak 61 was found in both parts of Merav, and peak 60 was detected in the fruit of both plants. Peak 59 had an oxidative molecular ion at *m/z* 609.1468 [M-2 H]– and a key aglycone fragment at *m/z* 301.0354 [M-H-308]^–^, indicating the loss of a rutinoside moiety, along with other oxidative fragments (299.1257 and 300.0286) and C-ring cleavage fragments (151.0025, 163.06029, and 271.0573). It was tentatively identified as delphinidin 3*-O-*(6ꞌꞌ*-O-*α-rhamnopyranosyl*-β*-glucopyranoside)^66^. Peak 61, found mainly in MVL, was identified as delphinidin aglycone^66^ with an oxidative ion at *m/z* 301.0749 [M-2 H]^–^. Peaks 62, 63, and 65 were classified as malvidin glycosides based on their aglycone fragments: peak 62 (*m/z* 493.1328 [M]^+^, 491.1202 [M–2 H]^–^) showed a fragment at *m/z* 329.0817 [M–H–162]^–^ due to galactose loss, identifying it as malvidin 3*-O-*galactoside; peak 65 (*m/z* 491.1162 [M–2 H]^–^) had a fragment at *m/z* 329.0668 [M–H–162]^–^ indicating glucoside loss, identified as malvidin 3*-O-*glucoside; and peak 63 (*m/z* 655.1688 [M]^+^) displayed fragments at *m/z* 493.1399 [M–162]^+^ and 331.2114 [M + 324]^+^, leading to identification as malvidin 3,5-di*-O-*glucoside^66^. Peak 64, presented molecular ions at *m/z* 461.1078 [M–2 H]^–^ and 463.1289 [M]^+^ and an aglycone fragment at *m/z* 299.8789 [M–H–162]^–^, corresponding to the loss of a hexose moiety, and was identified as peonidin 3*-O-*hexoside^66^.

#### Coumarins

Four coumarins were detected in all four extracts, as indicated in Table 3; two were detected at MTL, MVL and MTF, which were assigned to peaks 82 and 83, while peak 84 was detected in MTF only. The last was peak 85 which was detected in MVL, MTF and MVF. The characteristic fragmentation patterns of coumarins include the sequential loss of CO and/or CO2. Peak 82 was tentatively identified as scopoletin^67^ as it displayed a deprotonated molecular ion at *m/z* 191.0349, In its negative MS^2^ spectrum, the characteristic product ions were interpreted at *m/z* 176.0123 [M-H-CH3]^–^, and 148.1174 [M-H-CH3-CO]^–^. Peak 83 was assigned to daphnetin^68^ that showed a molecular ion peak at *m/z* 177.0566 [M-H]^–^. Its MS^2^ spectrum showed the aglycone ion at *m/z* 133.02689 [M–H– 44]^–^ corresponding to the loss of CO_2_. Peak 84 displayed a deprotonated ion at *m/z* 174.9582. In its MS^2^ spectrum, the characteristic product ions were interpreted at *m/z* 156.9518 [M-H-OH]^–^ and 130.5520 [M-H-CO_2_]^–^ that was in complete agreement with the library database and reported data of 7-hydroxy-4-methyl-coumarin^67^. Moreover, peak 85 was assigned to be di-hydroxycoumarin hexoside, where it showed a deprotonated molecular ion peak at *m/z* 339.2768 [M-H]^–^ and protonated ion *m/z* 341.1190 [M + H]^+^. Two distinctive product ions were visible in its negative MS^2^ spectra at *m/z* 177.1507 [M-H-162]^-^ and 131.0757 [M-H-162-44]^-^, which corresponded to the loss of the hexoside moiety and (hexoside + CO_2_), respectively. Based on published data, this metabolite was tentatively identified as esculin^42^.

#### Phenolic acids

Eight phenolic acids were detected across all four extracts, corresponding to peaks 69–75, and were primarily characterized by common fragmentation patterns including dehydration (-H₂O, 18 amu) and decarboxylation (-CO₂, 44 amu). Peak 69 showed a molecular ion at *m/z* 353.1095 [M–H]⁻ and was identified as chlorogenic acid^69^. Peak 70 displayed *m/z* 385.0780 [M–H]⁻ with an aglycone fragment at *m/z* 223.0448 [M–H–162]⁻ indicating the loss of a hexose and confirming it as 1*-O-β*-D-glucopyranosyl sinapate^70^. Peak 71, found in MVL and MVF, showed *m/z* 147.0397 [M–H]⁻ and a fragment at *m/z* 103.0584 [M–H–44]⁻, and was identified as trans-cinnamate^71^. Peak 72 appeared only in MTF with *m/z* 165.0587 [M + H]^+^ and a major fragment at *m/z* 120.9609 from decarboxylation, indicating *p*-coumaric acid. Peak 73 had a protonated ion at *m/z* 225.0932 with MS^2^ ions at *m/z* 210.1461, 195.0495, and 151.0554 corresponding to methyl losses and CO₂, confirming sinapic acid identity^72^. Peak 74, dominant in MVL showed *m/z* 179.0305 [M–H]⁻ with a fragment at *m/z* 135.0468 [M–H–44]⁻, aligning with caffeic acid^73^. Finally, peak 75 appeared only in MTL with *m/z* 359.1731 [M–H]⁻ and diagnostic fragments at *m/z* 197.1208 and 179.0981, along with further dehydrated and decarboxylated fragments at *m/z* 161.0424 and 135.0345, confirming it as rosmarinic acid^74^.

**Table 3:** Metabolites identified in the methanol extracts of Murcott leaves (MTL), Merav leaves (MVL), Murcott fruits (MTF), and Merav fruits (MVF) in positive ionization mode [M + H]^+^ and negative ionization mode [M-H]^−^.

Peak ID	Rt (min)	Compound name	MTL	MVL	MTF	MVF	Molecular formula	Error (ppm)	[M + H]^+^ (m/z )	MS^2^ Fragments (m/z )	[M-H]^−^ (m/z )	MS^2^ Fragments (m/z )
**Flavonoids**:												
• **Flavanones**												
1	6.530	Naringenin 7*-O-*glucoside	√	√	√	√	C_21_H_22_O_10_	9.6	435.1262	273.0559	433.185	151.0019, 271.0610
2	7.408	Eriodictyol 7*-O-*neohesperidoside	√	√	√	√	C_27_H_32_O_15_	0.1	−	−	595.1640	287.0571, 449.1302,
3	7.851	Isookanin 7-glucoside (Flavanomarein)	-	-	√	-	C_21_H_22_O_11_	-5.9	−	−	449.1027	269.0427, 287.0549,
4	8.197	Hesperetin 7*-O-*neohesperidoside	-	-	√	-	C_28_H_34_O_15_	4.1	−	−	609.1738	301.0613
5	8.438	Hesperidin	√	√	√	√	C_28_H_34_O_15_	-0.2	611.2517	303.11199	609.1814	301.0713
6	9.893	Isosakuranetin 7*-O-*neohesperidoside	√	√	√	√	C_28_H_34_O_14_	-3.1	595.2019	195.0290, 263.0552, 287.0906	593.1902	285.0609
7	11.061	Naringenin	√	√	√	√	C_15_H_12_O_5_	0.7	273.0745	121.0524, 153.02046,	271.0605	119.0498, 151.0036
8	11.485	Hesperetin	√	√	√	√	C_16_H_14_O_6_	1	303.0844	288.1534	301.0720	286.0478
9	18.860	5,7,3’,4’ tetrahydroxyflavanone	√	-	-	-	C_15_H_12_O_6_	-0.6	−	−	287.1661	251.0660, 269.2064
• **Flavones**												
10	5.742	Luteolin 7,3’-di*-O-*glucoside	√	-	-	√	C_27_H_30_O_16_	0.1	−	−	609.0918	283.09683
11	7.020	Luteolin 8*-C-*glucoside (Orientin)	√	-	√	-	C_21_H_20_O_11_	3.2	449.1772	329.09913	447.0936	327.0678, 357.0684
12	7.049	Luteolin 6*-C-*glucoside (Isoorientin)	-	√	√	√	C_21_H_20_O_11_	-0.1	449.1086	329.08692	447.0912	327.0698, 357.0664
13	7.292	Vitexin 2’’*-O-*rhamnoside	√	-	√	√	C_27_H_30_O_14_	5.8	−	−	577.1512	431.1573
14	7.496	Apigenin 8*-C-*glucoside (Vitexin)	√	√	√	√	C_21_H_20_O_10_	9	433.1492	285.1294, 343.1141	431.0981	283.0634, 311.0588, 341.0755
15	8.081	Rhoifolin (Apigenin 7*-O-*neohesperidoside)	√	√	√	√	C_27_H_30_O_14_	2.2	579.1668	271.0946	577.1533	269.0453
16	8.277	Diosmin	-	-	√	√	C_28_H_32_O_15_	1.3	609.1791	301.05007	−	−
17	8.438	Baicalein 7*-O-*glucuronide (Baicalin)	√	√	-	-	C_21_H_18_O_11_	2.6	−	−	445.1099	265.0979, 309.2156, 399.0928
18	8.476	Apigenin 7*-O-*glucoside	√	-	-	-	C_21_H_20_O_10_	6.2	−	−	431.0963	268.0388
19	9.031	Acacetin	-	√	√	-	C_16_H_12_O_5_	0.1	285.1271	270.9302	283.0620	196.0467, 239.1632, 247.14557, 268.0359
20	9.647	Acacetin 7*-O-*neohesperidoside	-	-	-	√	C_28_H_32_O_14_	1.6	−	−	591.1755	149.04949, 161.02716, 267.16993, 283.06346, 573.18067, 591.1755
21	9.731	Acacetin 7*-O-*rutinoside	√	-	√	√	C_28_H_32_O_14_	0.8	593.15	285.1078	591.3115	283.0629
22	9.909	Luteolin 4’*-O-*glucoside	-	-	√	√	C_21_H_20_O_11_	3.9	449.1597	287.0989	−	−
23	11.236	Apigenin	√	√	-	-	C_15_H_10_O_5_	6.7	−	−	269.0452	117.0344, 151.0038
24	13.789	Luteolin	√	√	√	√	C_15_H_10_O_6_	2.3	287.0881	153.1242	285.0768	151.0042
• **Isoflavones**												
25	1.155	Daidzein	-	-	-	√	C_15_H_10_O_4_	-9.5	255.1450	122.9401, 165.9184, 236.9890	−	−
26	4.679	Ononin	-	-	√	-	C_22_H_22_O_9_	-2	431.1562	226.9483, 269.1088,	−	−
27	5.483	Genistein	-	-	√	√	C_15_H_10_O_5_	5.9	271.1186	242.9196, 252.9485	−	−
28	5.755	Daidzein 8*-C-*glucoside	√	√	√	√	C_21_H_20_O_9_	-7.7	−	−	415.1227	371.1811
29	11.540	Sissotrin (5-hydroxy ononin)	-	-	√	√	C_22_H_22_O_10_	-5.8	447.1677	285.1139	−	−
30	12.672	Formononetin	-	√	-	-	C_16_H_12_O_4_	0.3	−	−	267.1938	195.0514, 211.0458, 224.0521, 252.046
• **Flavonols**												
31	0.984	Myricetin	√	√	-	√	C_15_H_10_O_8_	3.2	319.0969	124.9584	317.0550	122.9862, 273.00216,
32	1.362	Taxifolin	-	-	√	-	C_15_H_12_O_7_	-1	305.1297	287.1295	−	−
33	5.865	Kaempferol 3-Glucuronide	√	-	√	-	C_21_H_18_O_12_	-9.9	−	−	461.1724	285.09616, 415.19103
34	6.089	Kaempferol 7-neohesperidoside	√	√	√	√	C_27_H_30_O_15_	0.2	−	−	593.1493	285.0437
35	6.227	Quercetin 3-D-xyloside	√	-	-	-	C_20_H_18_O_11_	0.5	−	−	433.1754	301.10762
36	6.272	Gossypin	-	-	√	-	C_21_H_20_O_13_	2.9	481.1274	319.0977	−	−
37	6.278	Isorhamnetin 3*-O-*rutinoside	√	√	√	√	C_28_H_32_O_16_	4.6	625.1587	317.0988	623.1601	315.1159
38	6.419	Kaempferol 3*-O-*robinoside-7*-O-*rhamnoside	-	-	√	√	C_33_H_40_O_19_	-2.8	741.2368	433.1183, 595.1691	−	−
39	6.539	Quercetin 3*-O-*β-D-galactopyranoside (Hyperoside)	-	-	√	√	C_21_H_20_O_12_	0.7	465.1707	303.14627	−	−
40	6.610	Quercitrin	-	√	-	-	C_21_H_20_O_11_	3.3	−	−	447.1960	301.1678
41	6.618	Quercetin 3,4’*-O-*di-*β*-glucopyranoside	√	-	√	√	C_27_H_30_O_17_	-2.7	−	−	625.1803	301.0446
42	7.209	Kaempferol 3*-O-*α-L-rhamnoside	√	√	√	-	C_21_H_20_O_10_	1.4	−	−	431.1905	265.0933, 285.1485
43	7.362	Rutin	-	-	√	√	C_27_H_30_O_16_	4.3	611.1714	303.0598	−	−
44	7.662	Kaempferol 3*-O-*rutinoside	-	-	√	√	C_27_H_30_O_15_	2.5	595.1741	287.0784	−	−
45	7.886	Kaempferol 3*-O-*(6*-p-*coumaroyl)-glucoside	√	-	-	-	C_30_H_26_O_13_	0.5	−	−	593.1507	285.0403
46	7.972	Kaempferol 3*-O-*glucoside	-	-	-	√	C_21_H_20_O_11_	0	449.1830	287.1305	−	−
47	8.018	Myricitrin	√	-	-	-	C_21_H_20_O_12_	3.6	−	−	463.1173	317.0910
48	8.043	Syringetin 3*-O-*galactoside	√	√	√	-	C_23_H_24_O_13_	-0.9	−	−	507.1148	345.0618
49	8.088	Syringetin 3*-O-*glucoside	-	√	-	-	C_23_H_24_O_13_	2.6	−	−	507.1167	344.0563
50	8.342	Isorhamnetin 3*-O-*glucoside	-	√	√	-	C_22_H_22_O_12_	-3.2	−	−	477.1062	315.0541
51	8.424	Isoquercitrin	-	-	√	-	C_21_H_20_O_12_	-5.3	465.1398	303.0920	−	−
52	8.963	Quercetin 4’-glucoside	-	√	-	-	C_21_H_20_O_12_	7.6	−	−	463.2537	301.0728
53	11.260	Quercetin	√	-	√	-	C_15_H_10_O_7_	-6.2	303.1436	119.0423, 125.06459, 153.0238	301.0736	117.0197, 149.0611, 151.0038
54	11.534	3,5,7-trihydroxy-4’-methoxyflavone (Kaempferide)	√	√	√	√	C_16_H_12_O_6_	1.2	301.1431	286.0360	299.0562	284.0330
55	12.633	3’-methoxy-4’,5,7-trihydroxyflavonol (Isorhamnetin)	√	-	√	-	C_16_H_12_O_7_	8.3	317.0682	270.0245, 287.0478	315.0877	268.0450, 285.0424
56	14.076	3,5,3’,4’-tetrahydroxy-7-methoxyflavone	√	√	√	√	C_16_H_12_O_7_	-3.8	317.1238	231.9871, 249.0785,	315.0906	229.0174, 247.0620,
• **Flavanol**												
57	19.045	L-Epicatechin	√	√	-	-	C_15_H_14_O_6_	0.7	291.2321	205.9864, 247.2654	−	−
• **Aurones**												
58	5.625	Maritimetin 6*-O-*glucoside	-	-	√	√	C_21_H_20_O_11_	0.5	449.1412	287.08304	−	−
• **Anthocyanidins**												
59	7.382	Delphinidin 3*-O-*(6’’*-O-*α-rhamnopyranosyl-β-glucopyranoside)	√	√	√	√	C_27_H_31_O_16_	-5.5	−	−	609.1468 [M-2 H]^−^	151.0025, 163.06029, 271.0573, 299.1257, 300.0286, 301.0354,
60	7.414	Cyanidin 3*-O-*rutinoside (keracyanin)	-	-	√	√	C_27_H_31_O_15_	1.6	595.1634 [M]^+^	287.19833	−	−
61	7.882	Delphinidin	-	√	-	√	C_15_H_11_O_7_	-0.6	−	−	301.0749 [M-2 H]^−^	255.2276, 283.1827
62	8.085	Malvidin 3-galactoside	-	-	√	-	C_23_H_25_O_12_	-5.1	493.1328 [M]^+^	331.1293	491.1202 [M-2 H]^−^	329.0817, 330.9201
63	8.425	Malvidin 3, 5-di*-O-*glucoside chloride	-	-	√	-	C_29_H_35_O_17_	6.1	655.1688 [M]^+^	331.21144,	−	−
64	8.649	Peonidine 3*-O-*glucoside chloride	√	√	√	√	C_22_H_23_O_11_	-6.2	463.1289 [M]^+^	301.1236	461.1078 [M-2 H]^−^	299.8789
65	8.888	Malvidin 3*-O-*glucoside chloride	-	-	√	-	C_23_H_25_O_12_	0.2	−	−	491.1162 [M-2 H]^−^	329.0908
• **Chalcones**												
66	7.869	Okanin 4’*-O-*glucoside	-	√	-	√	C_21_H_22_O_11_	1.1	451.117	289.1178	449.0889	287.2334
67	8.888	Neohesperidin dihydrochalcone	-	-	√	-	C_28_H_36_O_15_	3.9	−	−	611.1354	303.0517, 345.0949, 405.0437, 449.0966, 465.1055, 491.1552
68	13.288	Phlorizin	√	-	√	-	C_21_H_24_O_10_	0.9	437.1937	275.0933	435.2028	273.1481
**Phenolic acid conjugates**												
69	0.972	Chlorogenic acid	√	-	√	-	C_16_H_18_O_9_	0.1	355.1735	181.0518, 193.0435,	353.1095	179.04203, 191.0895
70	1.945	1*-O-*b-D-glucopyranosyl sinapate	√	√	√	√	C_17_H_22_O_10_	-1.1	387.1826	225.9061	385.0780	223.04486
71	5.911	trans-Cinnamate	-	√	-	√	C_9_H_8_O_2_	5.5	−	−	147.0397	103.04567, 119.03396, 147.0438
72	5.961	3-(4-Hydroxyphenyl) prop-2-enoic acid (*p-* coumaric acid)	-	-	√	-	C_9_H_8_O_3_	0.8	165.0587	103.0584, 120.9609	−	−
73	6.361	3-(4-hydroxy-3,5-dimethoxyphenyl)-2-propenoic acid (sinapic acid)	-	-	√	√	C_11_H_12_O_5_	3.3	225.0932	151.0554, 195.0495, 210.1461	−	−
74	9.440	Caffeic acid	√	√	-	√	C_9_H_8_O_4_	4.5	181.1223	137.1266	179.0305	135.0468
75	10.040	Rosmarinic acid	√	-	-	-	C_18_H_16_O_8_	1.2	−	−	359.1731	135.0345, 161.0424, 179.0981, 197.1208
**Fatty acid and their conjugates**												
76	0.953	3-Hydroxy-3-Methylglutaric acid	-	√	-	-	C_6_H_10_O_5_	9.1	−	−	161.0429	117.1311, 143.0422
77	1.185	D-(+)-Malic acid	√	√	√	√	C_4_H_6_O_5_	0.7	−	−	133.014	89.0261, 115.0116
78	1.236	2-Isopropylmalic acid	√	√	√	-	C_7_H_12_O_5_	7.3	−	−	175.0592	131.0785, 157.0466,
79	15.542	4-(4-Hydroxy-2,6,6-trimethyl-1-cyclohexen-1-yl)-2-butanyl *β*-D-glucopyranoside	-	√	-	-	C_19_H_34_O_7_	0	397.2	212.1024, 233.1284	−	−
80	22.296	gamma-Linolenic acid	√	√	√	-	C_18_H_30_O_2_	1.6	−	−	277.2171	233.25313, 259.2215
81	23.662	3-ethyl-4-hydroxy-4-methylpentyl 6*-O-*[(2 S,3R,4R)-tetrahydro-3,4-dihydroxy-4-(hydroxymethyl)-2-furanyl] *β*-D-Glucopyranoside	-	√	-	-	C_19_H_36_O_11_	-4.7	463.2849 [M + Na]^+^	303.1417	−	−
**Coumarins**												
82	7.993	Scopoletin	√	√	√	-	C_10_H_8_O_4_	1.3	193.0473	133.0237, 150.0272, 178.0350	191.0349	148.0174, 176.0123
83	10.875	Daphnetin	√	√	√	-	C_9_H_6_O_4_	2.2	−	−	177.0566	133.02692
84	13.595	7-hydroxy-4-methylcoumarin	-	-	√	-	C_10_H_8_O_3_	5.5	−	−	174.9582	130.5520, 156.9518
85	16.441	Esculin	-	√	√	√	C_15_H_16_O_9_	2	341.1190	179.0878	339.2768	131.0757, 177.15072
**Nitrogenous compounds**												
86	1.273	1-methylpyridinio-3-carboxylate	-	-	√	-	C_7_H_7_NO_2_	0	138.0528	80.0437, 94.0741	−	−
87	6.073	Ferrioxamine	-	√	-	-	C_27_H_45_FeN_6_O_9_	-0.9	654.2676	601.2931, 636.2343	−	−
88	21.887	Octocrylene	-	√	-	-	C_24_H_27_NO_2_	-0.2	362.213	291.2485, 317.1709	−	−
**Terpenes and terpenoids**												
89	12.222	(6,6-Dimethylbicyclo[3.1.1] hept-2-yl)methyl 6*-O-*[(2R,3R,4R)-3,4-dihydroxy-4-(hydroxymethyl)tetrahydro-2-furanyl]-*β*-D-glucopyranoside	√	√	-	-	C_21_H_36_O_10_	-0.7	471.1636 [M + Na]^+^	289.9070, 307.1324	−	−
90	14.167	4-[4-(*β*-D-glucopyranosyloxy)-2-hydroxy-2,6,6-trimethylcyclohexylidene]-3-Buten-2-one	√	√	-	-	C_19_H_30_O_8_	0.5	409.1627 [M + Na]^+^	245.1423	−	−
91	14.781	1-Acetoxy-7-isopropylidene-1,4a-dimethyl-6-oxodecahydro-2-naphthalenyl 2,3-dimethyl-2-oxiranecarboxylate	√	√	-	-	C_22_H_32_O_6_	0.9	415.2122 [M + Na]^+^	306.9146, 350.1746	−	−
92	15.530	7b,9-Dihydroxy-3-(hydroxymethyl)-1,1,6,8-tetramethyl-5-oxo-1,1a,1b,4,4a,5,7a,7b,8,9-decahydro-9aH-cyclopropa[3,4]benzo[1,2-e]azulen-9a-yl acetate	√	√	-	-	C_22_H_30_O_6_	2.2	432.237 [M + ACN + H]^+^	354.1479, 372.1671	−	−
93	15.530	3-(acetoxymethyl)-5-(2-formyl-4-hydroxy-5,5,8a-trimethyl-1,4,4a,6,7,8-hexahydronaphthalen-1-yl)pent-2-enoic acid	√	√	-	-	C_22_H_32_O_6_	-1.1	415.2124 [M + Na]^+^	355.2124	−	−
94	17.544	Hetisine	√	√	-	-	C_20_H_27_NO_3_	-1	330.338	286.2968, 295.2156	−	−
95	20.281	1-Naphthalenepentanol, decahydro-2-hydroxy-gamma,2,5,5,8a-pentamethyl-, α-acetate	-	√	-	-	C_22_H_40_O_3_	-3.4	375.1954 [M + Na]^+^	275.18255,	−	−
**Miscellaneouse compounds**												
96	0.897	Mucate	√	√	-	-	C_6_H_10_O_8_	0.3	−	−	209.0296	165.0320, 191.0381
97	0.972	E-3,4,5’-Trihydroxy-3’-glucopyranosylstilbene	√	√	√	√	C_20_H_22_O_9_	-0.6	407.1649	245.1125	405.1029	243.0556
98	1.311	D-(-)-Quinic acid	√	√	√	√	C_7_H_12_O_6_	-6.5	−	−	191.0577	127.04993, 173.94166
99	1.590	Resveratrol	-	√	√	√	C_14_H_12_O_3_	-3.2	229.0318	145.09193,187.97542	227.1308	143.07954, 185.1308
100	12.214	Benzyl 6*-O-β*-D-glucopyranosyl-*β*-D-glucopyranoside	√	√	-	-	C_19_H_28_O_11_	-0.4	455.1904 [M + Na]^+^	293.8922	−	−
101	13.807	2-Phenylethyl 2*-O-*[(2 S,3R,4R)-3,4-dihydroxy-4-(hydroxymethyl)tetrahydro-2-furanyl]-*β*-D-glucopyranoside	√	-	-	-	C_19_H_28_O_10_	-2.7	439.1969 [M + Na]^+^	275.0986	−	−
102	14.121	Phenylmethyl 6*-O-*[(2R,3R,4R)-tetrahydro-3,4-dihydroxy-4-(hydroxymethyl)-2-furanyl]-*β*-D-glucopyranoside	-	√	-	-	C_18_H_26_O_10_	-0.1	425.1375	261.9109	−	−
103	14.716	Cascaroside D	√	-	-	-	C_27_H_32_O_13_	-0.9	587.2112 [M + Na]^+^	407.1388, 425.1842,	−	−
104	15.507	1,5-Anhydro-2*-O-*(6*-O-*benzoyl-α-D-galactopyranosyl)-D-glucitol	√	√	-	-	C_19_H_26_O_11_	3.9	453.1649 [M + Na]^+^	289.2938	−	−
105	21.584	Avobenzone	√	√	-	-	C_20_H_22_O_3_	-0.2	311.2564	219.1414, 266.2041	−	−
106	22.076	(2,6,6-Trimethyl-4-oxo-2-cyclohexen-1-yl)methyl *β*-D-glucopyranoside	√	√	-	-	C_16_H_26_O_7_	-3.9	353.2682 [M + Na]^+^	191.0987	−	−
107	25.607	2-Acetoxy-4-pentadecylbenzoic acid	-	√	-	-	C_24_H_38_O_4_	-0.1	413.269 [M + Na]^+^	351.2435, 395.2117,	−	−

### Body weight evaluation

Body weight was tracked biweekly and displayed in Table 4. The Letrozole + HFD-treated mice experienced a notable weight gain of 12.76% compared to the control group. In contrast, the body weight of HFD-treated mice given Murcott and Merav fruit and leaf extracts (500 mg each) and metformin (300 mg) individually decreased by 7.89%, 14.51%, 10.53%, 24.41%, and 27.63%, respectively, compared to PCOS-induced mice (*p* < 0.05).

Although specific toxicity assessments were not part of the current study, no adverse behavioral changes or mortality were observed during the experimental period. Future investigations will incorporate detailed acute and sub-chronic toxicity studies to ensure safety and determine the therapeutic index.

**Table 4:** Changes in body weight control and experimental groups of mice.

No.	Groups	Number of weeks Body weight of mice(g)				
		0	2	4	6	8
(I)	Normal control	35.23 ± 0.36	36.40 ± 0.52	37.96 ± 0.69	38.80^A^ ± 3.68	40.65 ^AB^ ± 1.31
(II)	PCOS-induced group [HFD + Letrozole (90 µg /kg.b.w.)]	34.69 ± 0.377	36.88 ± 0.96	40.29 ^a AB^ ± 1.39	45.43 ^a ABC^ ± 2.69	51.93 ^a ABCD^ ± 2.012
(III)	PCOS + MTF (500 mg/kg.b.w.)	34.72 ± 0.92	35.193 ± 0.87	35.32 ^b^ ± 0.61	41.38 ^ab A^ ± 0.511	47.83 ^ab AD^ ± 2.08
(IV)	PCOS + MTL (500 mg/kg.b.w.)	34.62 ± 1.08	35.32 ± 1.24	36.29 ^b^ ± 0.88	38.19 ^bc^ ± 1.03	44.39 ^abc^ ± 1.16
(V)	PCOS + MVF (500 mg/kg.b.w.)	37.50 ± 0.60	36.04 ± 0.46	37.70 ^b^ ± 0.90	40.12 ^d A^ ± 0.40	46.46 ^ab ABCD^ ± 0.98
(VI)	PCOS + MVL (500 mg/kg.b.w.)	35.92 ± 1.48	34.08 ± 1.66	35.93 ^b^ ± 0.25	36.16 ^bce^ ± 0.25	39.25 ^abcde ABCD^ ± 0.65
(VII)	PCOS + Metformin (300 mg/kg.b.w.)	35.50 ± 0.71	33.63 ± 1.677	35.25 ^b^ ± 1.35	37.72 ^bce^ ± 0.98	37.72 ^abcde^ ± 0.98
	F	1.28^*^	5.76^*^	11.93^*^	16.43^*^	21.74^*^
	*p*	< 0.05^*^	< 0.05^*^	< 0.05^*^	< 0.05^*^	< 0.05^*^

Body weight of mice consuming regular diet, high fat diet + Letrozole (90 µg /kg.b.w.), high fat diet + Letrozole (90 µg /kg.b.w.) plus different extracts (500 mg/kg) and metformin (300 mg/kg.b.w.) during the last three weeks. Values are given as mean ± SD significantly different at *P* ≤ 0.05 for groups of six animals each. Small letters are used for comparison between the means within the column. Capital letters are used to compare means within the row. a: Significant with Group I, b: Significant with Group II, c: Significant with Group III, d: Significant with Group IV, e: Significant with Group V, f: Significant with Group VI, Abbreviation: Murcott Fruit (MTF), Murcott Leaves (MTL), Merav Fruit (MVF), and Merav Leaves (MVL).

### Effect of Murcott and Merav on plasma lipid profile in PCOS-induced mice

In Letrozole-treated mice on a high-fat diet, plasma total cholesterol (TC) and triglyceride (TG) levels rose significantly by 51.28% and 91.44%, respectively, while plasma HDL-C levels dropped by 58.70% (Table 5). Compared to Letrozole + HFD-treated mice, plasma TC and TG levels in PCOS-induced mice treated with MTF, MTL, MVF and MVL extracts (500 mg) were significantly lower (*p* < 0.05) by (20.35 and 12.06%), (26.50 and 26.21%), (23.61 and 19.71%), and (30.26 and 32.44%), respectively. Additionally, HDL-C levels were significantly higher by 86.94, 126.67, 106.42 and 170.67% (*p* < 0.05). Treatment with metformin (300 mg/kg.b.w) also significantly reduced TG and TC levels by 33.15% and 36.51%, respectively, and increased HDL-C levels by 178.21% (*p* < 0.05) compared to PCO-induced mice.

**Table 5:** Impact of Murcott and Merav fruits and leaves on plasma lipid profile in PCOS-induced mice.

No.	Group	TC (mg/dl)	TG (mg/dl)	HDL-c (mg/dl)
I	Normal control	157.56 ± 3.03	92.19 ± 3.50	43.68 ± 2.28
II	PCOS-induced group [HFD + Letrozole (90 µg /kg.b.w.)]	238.36 ^a^ ± 14.81	176.49 ^a^ ± 9.58	14.32 ^a^ ± 1.304
III	PCOS + MTF (500 mg/kg.b.w.)	189.83 ^ab^ ± 2.79	155.17 ^ab^ ± 3.78	26.77 ^ab^ ± 1.10
IV	PCOS + MTL (500 mg/kg.b.w.)	175.18 ^ab^ ± 5.36	132.44 ^abc^ ± 6.91	32.46 ^abc^ ± 0.61
V	PCOS + MVF (500 mg/kg.b.w.)	182.06 ^ab^ ± 5.40	141.69 ^abcd^ ± 3.46	29.56 ± 1.20 ^ab^
VI	PCOS + MVL (500 mg/kg.b.w.)	166.23 ^abc^ ± 10.55	119.23 ^abcde^ ± 8.39	38.76 ± 2.95 ^abcde^
VII	PCOS + Metformin (300 mg/kg b.w)	159.33 ^abcd^ ± 7.33	112.04 ^abcde^ ± 9.66	39.84 ± 1.13 ^abcde^
	F	69.83^*^	84.62^*^	127.8^*^
	*p*	< 0.05^*^	< 0.05^*^	< 0.05^*^

Data shown mean ± standard deviation of number of observations within each treatment. F: for One way ANOVA test, pairwise comparison between each group was done using Post Hoc Test (Tukey). *: Statistically significant at *p* ≤ 0.05.

a: Significant with Group I b: Significant with Group II c: Significant with Group III.

d: Significant with Group IV e: Significant with Group V f: Significant with Group VI.

Abbreviation: Murcott Fruit (MTF), Murcott Leaves (MTL), Merav Fruit (MVF), and Merav Leaves (MVL).

### Effect of Murcott and Merav on ovarian oxidative stress markers in PCOS-induced mice

Table 6 illustrates the levels of ovarian GSH, SOD, CAT, and MDA in normal and PCOS-induced mice treated with MTF, MTL, MVF and MVL extracts. Compared to normal mice (Group I), there was a significant decrease (*p* < 0.05) in ovarian GSH, SOD, and CAT levels by 64.19%, 160.89%, and 52.60%, respectively, and a significant increase in ovarian MDA by 54.28% after 8 weeks of Letrozole + HFD administration. Mice receiving MTF (500 mg/kg.b.w.) showed 59.50%, 45.79%, and 37.78% higher ovarian GSH, SOD, and CAT levels, respectively, and a significant decrease in ovarian MDA by 23.18% compared to PCOS-induced mice (*p* < 0.05). Treatment with MTL (500 mg/kg.b.w.) led to a significant increase in GSH, SOD, and CAT levels by 103.08%, 78.41%, and 57.01%, respectively, and a 29.21% reduction in MDA levels in comparison to the PCOS-induced mice (*p* < 0.05). Mice receiving MVF (500 mg) showed significant increases in GSH, SOD, and CAT levels by 69.28%, 58.32%, and 47.51%, respectively, and a decrease in MDA levels by 26.83% relative to PCOS-induced mice after 8 weeks (*p* < 0.05). Mice receiving MVL (500 mg) showed significant increases in GSH, SOD, and CAT levels by 139.95%, 211.88%, and 83.67%, respectively, and a decrease in MDA levels by 33.20% relative to PCOS-induced mice after 8 weeks (*p* < 0.05). Additionally, mice receiving Metformin (300 mg) showed significant increases in GSH, SOD, and CAT levels by 75.59%, 47.52%, and 28.05%, respectively, and a decrease in MDA levels by 18.03% relative to PCOS-induced mice after 8 weeks (*p* < 0.05).

**Table 6:** Impact of Murcott and Merav fruits and leaves on ovarian oxidative stress markers in PCOS-induced mice.

No.	Group	GSH (mg/g tissue)	SOD (IU/g tissue)	CAT (IU/g tissue)	MDA (nmol/mg tissue)
I	Normal control	9.97 ± 0.43	11.84 ± 0.73	36.43 ± 1.78	198.8 ± 12.15
II	PCOS-induced group [HFD + Letrozole (90 µg /kg.b.w)]	3.58^a^ ± 0.25	4.63^a^ ± 0.27	17.26^ab^ ± 1.21	306.7^ab^ ± 12.88
III	PCOS + MTF (500 mg/kg.b.w)	5.71 ^ab^ ± 0.36	6.75 ^ab^ ± 0.13	23.78^ab^ ± 2.70	235.6^ab^ ± 9.75
IV	PCOS + MTL (500 mg/kg.b.w)	7.27 ^abc^ ± 0.59	8.26 ^abc^ ± 0.36	27.10^ab^ ± 1.17	217.1^b^ ± 16.62
V	PCOS + MVF (500 mg/kg.b.w)	6.60 ^abc^ ± 0.33	7.33 ^abd^ ± 0.44	25.46^ab^ ± 2.52	224.4^ab^ ± 6.11
VI	PCOS + MVL (500 mg/kg.b.w)	8.59 ^abcde^ ± 0.43	9.81 ^abcde^ ± 0.36	31.70^abcde^ ± 2.86	204.8^bc^ ± 9.99
VII	PCOS + Metformin (300 mg/kg.b.w)	6.25 ^abdf^ ± 0.49	6.83 ^abdf^ ± 0.55	22.10^abdf^ ± 2.48	251.4^abdef^ ± 13.98
	F	138.7^*^	168.1^*^	49.19^*^	55.46*
	*p*	< 0.05^*^	< 0.05^*^	< 0.05^*^	< 0.05*

Data shown mean ± standard deviation of number of observations within each treatment. F: for One way ANOVA test, pairwise comparison between each group was done using Post Hoc Test (Tukey). *: Statistically significant at *p* ≤ 0.05.

a: Significant with Group I, b: Significant with Group II, c: Significant with group III, d: Significant with Group IV, e: Significant with Group V, f: Significant with Group VI, Abbreviation: Murcott Fruit (MTF), Murcott Leaves (MTL), Merav Fruit (MVF), and Merav Leaves (MVL).

### Effect of Murcott and Merav on plasma FSH, LH and testosterone levels in PCOS-induced mice

Table 7 shows significant changes in ovarian hormone levels in normal and treated mice. In PCOS-induced mice, ovarian FSH decreased by 67%, while testosterone and LH levels increased by 1204.77% and 181.66%, respectively. Treatment with MTF significantly increased ovarian FSH by 93.79% and reduced testosterone and LH levels by 77.46% and 41.08%, respectively (*p* < 0.05). MTL treatment increased ovarian FSH by 122.98% and reduced testosterone and LH levels by 90.32% and 54.41%, respectively (*p <* 0.05). MVF treatment increased ovarian FSH by 101.63% and reduced testosterone and LH levels by 86.09% and 45.11%, respectively (*p <* 0.05). MVL treatment increased ovarian FSH by 175.41% and reduced testosterone and LH levels by 92.51% and 68%, respectively (*p* < 0.05). Metformin treatment increased ovarian FSH by 117.02% and reduced testosterone and LH levels by 76.27% and 59.69%, respectively (*p <* 0.05).

**Table 7:** Impact of Murcott and Merav fruits and leaves on plasma FSH, LH and testosterone levels in PCOS-induced mice.

No.	Group	FSH (mIU/ml)	Testosterone (nmol/ml)	LH (mIU/ml)
I	Normal control	11.45 ± 0.78	0.84 ± 0.06	2.29 ± 0.05
II	PCOS-induced group [HFD + Letrozole (90 µg /kg.b.w.)]	3.70^ab^ ± 0.29	10.96^ab^ ± 0.36	6.45^ab^ ± 0.24
III	PCOS + MTF (500 mg/kg.b.w)	7.17^ab^ ± 0.43	2.47^ab^ ± 0.21	3.80^ab^ ± 0.29
IV	PCOS + MTL (500 mg/kg.b.w)	8.25^abc^ ± 0.50	1.09^bc^ ± 0.11	2.94^abc^ ± 0.22
V	PCOS + MVF (500 mg/kg.b.w)	7.46^ab^ ± 0.56	1.53^abcd^ ± 0.19	3.54^abd^ ± 0.24
VI	PCOS + MVL (500 mg/kg.b.w)	10.19^bcde^ ± 0.75	0.82^bce^ ± 0.09	2.03^bcde^ ± 0.17
VII	PCOS + Metformin (300 mg/kg b.w)	8.03^abf^ ± 0.59	2.60^abdef^ ± 0.20	2.60^bcef^ ± 0.20
	F	116.8^*^	2034^*^	290.7^*^
	*p*	< 0.05^*^	< 0.05^*^	< 0.05^*^

Data shown mean ± standard deviation of number of observations within each treatment. F: for One way ANOVA test, pairwise comparison between each group was done using Post Hoc Test (Tukey). *: Statistically significant at *p* ≤ 0.05.

a: Significant with Group I, b: Significant with Group II, c: Significant with Group III, d: Significant with Group IV, e: Significant with Group V, f: Significant with Group VI, Abbreviation: Murcott Fruit (MTF), Murcott Leaves (MTL), Merav Fruit (MVF), and Merav Leaves (MVL).

### Effect of Murcott and Merav on plasma COX-2 and ovarian NF-kβ levels in PCOS-induced mice

In PCO-induced animals, ovarian COX-2 and NF-kβ levels were markedly increased by 437.15% and 178.11%, respectively, compared to control mice (*p* < 0.05) (Table 8). MTF (500 mg/kg.b.w.) treatment significantly decreased these levels by 53.25% and 36.74%, respectively, compared to the PCO-induced group (*p* < 0.05). MTL treatment resulted in even larger reductions in ovarian COX-2 and NF-kβ levels, showing decreases of 66.13% and 56.16%, respectively, compared to the PCOS group. MVF treatment also significantly reduced ovarian COX-2 and NF-kβ levels by 75.82% and 43.04%, respectively, compared to the PCOS-induced mice. MVL supplementation brought about the most notable reductions, with ovarian COX-2 and NF-kβ levels declining by 81.50% and 69.02%, respectively, compared to the PCOS group (*p* < 0.05). Lastly, Metformin treatment significantly lowered ovarian COX-2 and NF-kβ levels by 44.80% and 30.97%, respectively, compared to the PCOS-induced group (*p* < 0.05).

**Table 8:** Impact of Murcott and Merav fruits and leaves on plasma COX-2 and ovarian NF-kβ levels in PCOS-induced mice.

No.	Group	COX-2 (U/ml)	NF-kβ (ng/mg)
I	Normal control	3.15 ± 0.30	1.37 ± 0.187
II	PCOS-induced group [HFD + Letrozole (90 µg /kg.b.w.)]	16.92^ab^ ± 1.40	3.81^a^ ± 0.15
III	PCOS + MTF (500 mg/kg.b.w)	7.91^ab^ ± 0.65	2.41^ab^ ± 0.21
IV	PCOS + MTL (500 mg/kg.b.w)	5.73^abc^ ± 0.29	1.67^bc^ ± 0.13
V	PCOS + MVF (500 mg/kg.b.w)	4.09^bcd^ ± 0.34	2.17^adb^ ± 0.24
VI	PCOS + MVL (500 mg/kg.b.w)	3.13^bcd^ ± 0.13	1.18^bcde^ ± 0.15
VII	PCOS + Metformin (300 mg/kg b.w)	9.34 ^abcdef^ ± 0.78	2.63^abdef^ ± 0.11
	F	307.9^*^	162.1^*^
	*p*	< 0.05^*^	< 0.05^*^

The data are presented as the mean ± standard deviation based on the number of observations for each treatment. F: for One way ANOVA test, pairwise comparison between each group was done using Post Hoc Test (Tukey). *: Statistically significant at *p* ≤ 0.05.

a: Significant with Group I, b: Significant with Group II, c: Significant with group III, d: Significant with Group IV, e: Significant with Group V, f: Significant with Group VI, Abbreviation: Murcott Fruit (MTF), Murcott Leaves (MTL), Merav Fruit (MVF), and Merav Leaves (MVL).

### Effect of Murcott and Merav on ovarian miR-33b-5P and miR-145 gene expression in PCOS-induced mice

**Fig. 3:**
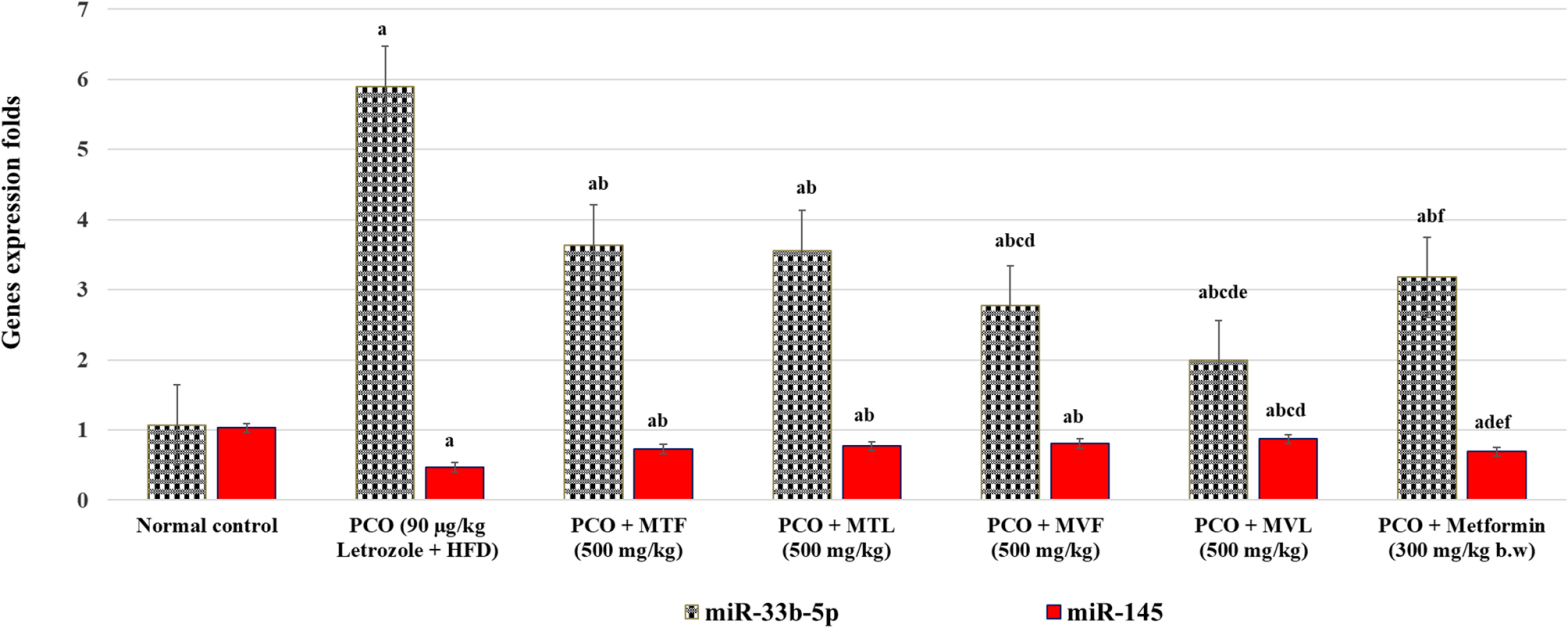
Impact of Murcott and Merav fruits and leaves on ovarian nuclear miR-33b-5P and miR-145 gene expression in mice. The data are presented as the mean ± standard deviation based on the number of observations for each treatment. F: F for One way ANOVA test, pairwise comparison between each groups was done using Post Hoc Test (Tukey). *: Statistically significant at *p* ≤ 0.05. a: Significant with Group I, b: Significant with Group II, c: Significant with Group III, d: Significant with Group IV, e: Significant with Group V, f: Significant with Group VI. Murcott Fruit (MTF), Murcott Leaves (MTL), Merav Fruit (MVF), and Merav Leaves (MVL).

Figure 3 shows the expression levels of ovarian miR-33b-5P and miR-145 genes in normal and PCOS-induced mice. Compared to normal mice, miR-33b-5P was significantly higher by 451.41%, while miR-145 was downregulated by 54.36% in Letrozole + HFD-administered mice. In comparison to PCOS-induced mice, MTF (500 mg/kg b.w.) treatment significantly reduced ovarian miR-33b-5P expression by 38.42% and significantly increased miR-145 gene expression by 55.32% (*p* < 0.05). Similarly, MTL (500 mg/kg b.w.) treatment resulted in a significant decrease in ovarian miR-33b-5P by 39.66% and a significant increase in miR-145 gene expression by 63.84% (*p* < 0.05). MVF (500 mg/kg b.w.) administration resulted in a notable reduction in ovarian miR-33b-5P by 53.05% and a significant upregulation of miR-145 gene expression by 72.34% (*p <* 0.05). MVL treatment significantly decreased ovarian miR-33b-5P by 66.27% and significantly upregulated miR-145 gene expression by 85.11% (*p <* 0.05). Furthermore, Metformin treatment resulted in substantial reductions, with ovarian miR-33b-5P decreasing by 46.10% and miR-145 gene expression increasing by 45.80%, compared to PCOS-induced mice (*p <* 0.05). All the previous data were shown in Fig. 3.

### Histological examination of mice ovarian tissues of targeted groups compared to control group

**Fig. 4:**
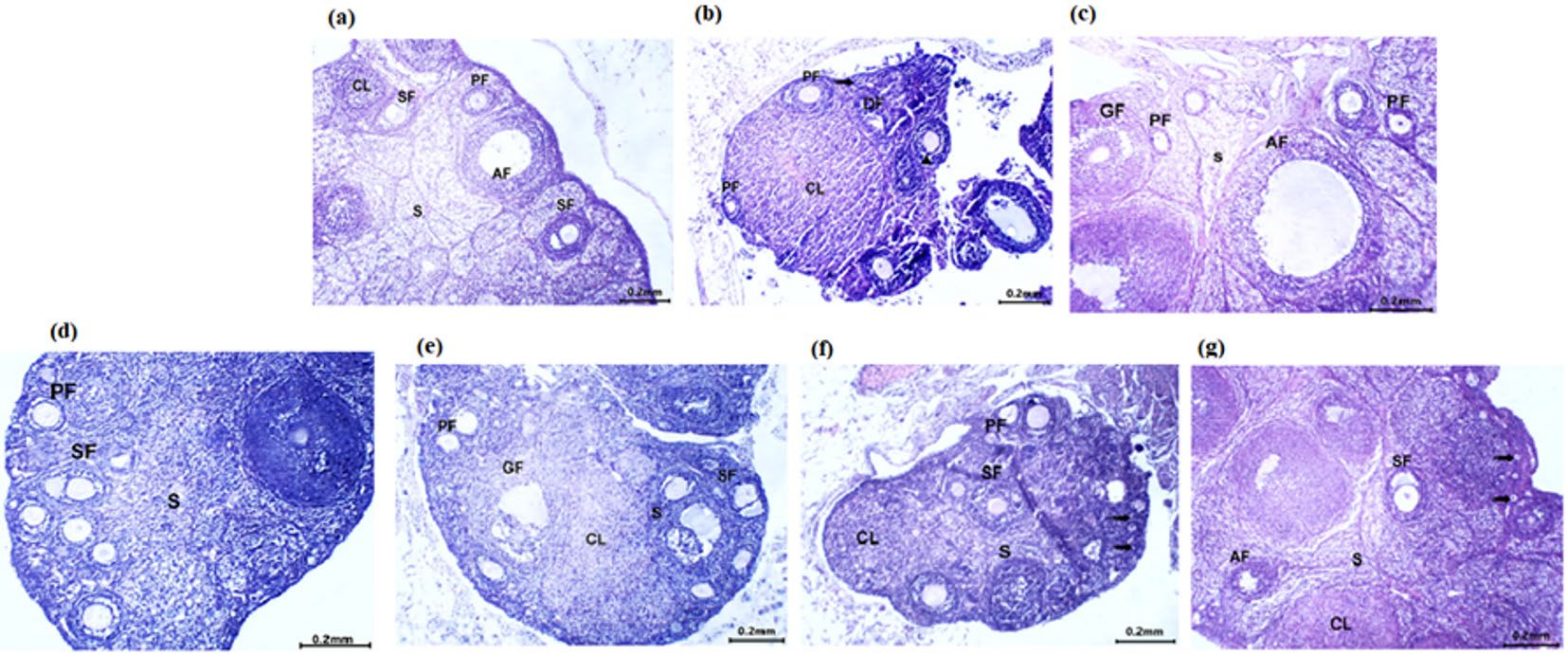
Histological analysis of ovarian tissue sections from mice, stained with hematoxylin and eosin (H&E; 200x), was performed for various groups and compared to the control group. (**a**): Group I: Normal control; (**b**): Group II: PCOS group, Was administrative Letrozole (90 µg/kg) and fed HFD; (**c**): Group III: PCO induced mice treated with MTF(500 mg/kg); (**d**): Group IV: PCO induced mice treated with MTL(500 mg/kg); (**e**): Group V: PCO induced mice treated with MVF(500 mg/kg); (**f**): Group VI: PCO induced mice treated with MVL(500 mg/kg); and (**g**): Group VII: PCO induced mice treated with Metformin (300 mg/kg b.w). Primary follicles (PF), secondary follicles (SF), corpora lutea (CL). MTL follicles displayed nuclei that were strongly stained (bifid arrow), primordial follicles (dotted arrow), normal stroma (S), atretic follicles (AF) and degenerated follicles (DF).

In the control group, ovarian tissues showed normal histology, with a cortex containing primordial follicles (single layer of flattened cells), primary follicles (one or multiple layers of cuboidal granulosa cells), secondary follicles (with multilayered granulosa cells, oocyte, and antral cavities), and a healthy stroma. The outer cortex also displayed corpora lutea with foamy acidophilic cytoplasm and pale nuclei, along with atretic follicles and mature Graafian follicles characterized by organized granulosa layers, liquid folliculi, corona radiata, zona pellucida, and a fibrous theca externa. In contrast, PCOS-induced mice exhibited severe ovarian damage, including degenerated follicles with black pyknotic nuclei, disorganized and vacuolated granulosa cells, a significant reduction in primary follicles, very few corpora lutea, absence of primary and secondary follicles, and extensive stromal cell infiltration. Treatment with MTF (500 mg/kg) successfully restored normal ovarian architecture, with various developing follicles and small clusters of large lutein cells in the corpus luteum. MTL-treated mice showed multilaminar primary and secondary follicles, normal stroma, and some follicles with darkly stained nuclei. MVF treatment effectively reestablished typical ovarian structure, featuring mature Graafian follicles, numerous primary follicles, few degenerated follicles, and large corpora lutea with mildly eosinophilic cytoplasm. Similarly, MVL treatment resulted in ovarian tissue closely resembling the control group, with a defined cortex and medulla containing primordial, primary, secondary, and few atretic follicles, alongside well-formed corpora lutea and a healthy stroma. Finally, metformin treatment at 300 mg/kg also promoted recovery, as shown by the presence of multiple developing follicles, corpora lutea, and normal stromal architecture, despite occasional strongly stained nuclei. Overall, these treatments, especially at higher doses, effectively reversed PCOS-induced ovarian damage and restored normal histological features. As illustrated in Fig. 4.

## Conclusion

This study highlights the therapeutic potential of Murcott and Merav (*Citrus reticulata*) fruit and leaf extracts in managing polycystic ovary syndrome (PCOS) through their ability to restore hormonal balance, reduce oxidative stress, improve lipid profiles, regulate inflammatory markers, and correct ovarian tissue morphology. In the PCOS-induced model, letrozole combined with a high-fat diet resulted in significant metabolic and histological alterations, consistent with typical PCOS pathology, including increased body weight, dyslipidemia, elevated testosterone and LH levels, suppressed FSH, oxidative stress, and inflammation, alongside ovarian structural damage with follicular degeneration and stromal infiltration.

Treatment with Murcott and Merav extracts (MTF, MTL, MVF, MVL) at 500 mg/kg markedly reversed these pathological features. Particularly, MVL demonstrated the most potent therapeutic effects, significantly improving body weight, reducing cholesterol and triglyceride levels, increasing HDL-C, enhancing antioxidant defense (GSH, SOD, CAT), and reducing oxidative marker MDA. MVL also achieved the most substantial reduction in inflammatory mediators COX-2 and NF-kβ. On the molecular level, MVL upregulated miR-145 expression and downregulated miR-33b-5P more effectively than the other treatments, highlighting its role in modulating oxidative stress and inflammation at the genetic level.

The observed modulation of LH, FSH, and testosterone levels reflects a restoration of the hypothalamic–pituitary–ovarian (HPO) axis, which is typically disrupted in PCOS. Elevated LH and testosterone, along with reduced FSH, are hallmarks of PCOS and contribute to anovulation and follicular arrest. The extracts’ ability to normalize these hormones suggests a rebalancing of gonadotropin secretion and androgen synthesis. Furthermore, the significant downregulation of COX-2 and NF-kβ indicates suppression of chronic inflammation, a key driver of ovarian dysfunction in PCOS. The upregulation of miR-145 and downregulation of miR-33b-5P further support this anti-inflammatory and antioxidative effect, as these miRNAs are known to regulate genes involved in lipid metabolism, oxidative stress, and inflammatory signaling. miR-145, in particular, has been shown to promote granulosa cell function and follicular development, while miR-33b-5P is associated with lipid dysregulation and inflammation. Thus, the extracts may exert their therapeutic effects by targeting both endocrine and molecular pathways central to PCOS pathogenesis.

Histologically, treatment with MTF, MTL, MVF, and MVL restored normal ovarian architecture, with the reappearance of various stages of developing follicles and corpora lutea, reduction of atretic and degenerated follicles, and normalization of the stromal tissue. MVL treatment achieved the most complete histological recovery, closely resembling the normal control group. Compared to standard treatment with metformin (300 mg/kg), the citrus extracts, particularly MVL, showed comparable or superior effects across most parameters, suggesting that natural bioactive compounds, especially methylated flavonoids such as methylated kaempferol present in Merav leaves, might offer effective and safer alternatives for PCOS management. These findings support previous research showing that flavonoids and other phenolic compounds have powerful antioxidant, anti-inflammatory, and hormone-regulating properties, making them potential therapeutic options for PCOS. Thus, the Murcott and Merav varieties, particularly the Merav leaf extract, represent promising natural therapeutic agents for mitigating PCOS symptoms, improving reproductive health, and addressing associated metabolic and inflammatory disturbances.

### Study limitations

A limitation of the current study is the use of a single dose (500 mg/kg b.w.) for evaluating the therapeutic effects of the extracts. While this dose was selected based on literature precedent and demonstrated significant efficacy, future studies should include multiple dose levels to establish a clearer dose-response relationship. This would enhance the pharmacological relevance of the findings and support more precise therapeutic recommendations. On the other hand, the combination of HFD and letrozole has been widely used to induce both metabolic and reproductive features of PCOS, including hyperandrogenism and ovarian dysfunction. We acknowledge that insulin resistance was not evaluated through markers such as fasting insulin, glucose tolerance, or HOMA-IR. Future studies are warranted to include these parameters to better characterize the metabolic phenotype of the model and to explore whether the tested extracts exert beneficial effects on insulin sensitivity.

## Supplementary Information

Below is the link to the electronic supplementary material.

Supplementary Material 1

## Data Availability

All data analyzed during this study are presented and included in this article and supplementary file.
