# Supplementary material for: Metabolic identification of bioactive compounds of Citrus reticulata cultivars extracts for a novel approach to polycystic ovary syndrome
Source: Sci Rep. 2025 Sep 12;15:32454. doi: 10.1038/s41598-025-18116-5 (PMC12432226; doi:10.1038/s41598-025-18116-5)
Supplement: Supplementary file 1 — Supplementary Material 1 [file 41598_2025_18116_MOESM1_ESM.docx]

Supplementary data

|    |
| --- |
|  |
|  |
| **Fig.1. XIC chromatogram and MS spectra of Naringenin (Negative ionization mode)** |
|    |
|  |
|  |
| **Fig.2. XIC chromatogram and MS spectra of Naringenin7*-O-*hexoside (Negative ionization mode)** |

| 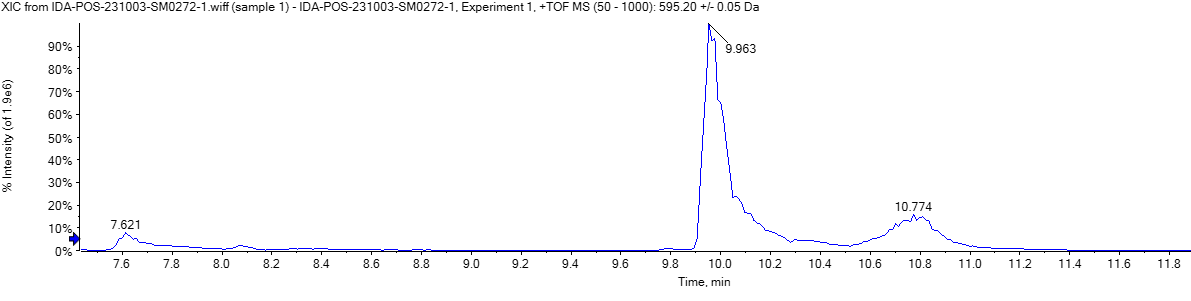  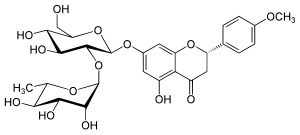 |
| --- |
| **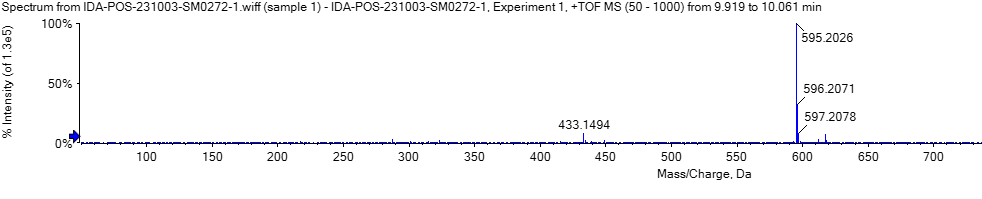** |
| **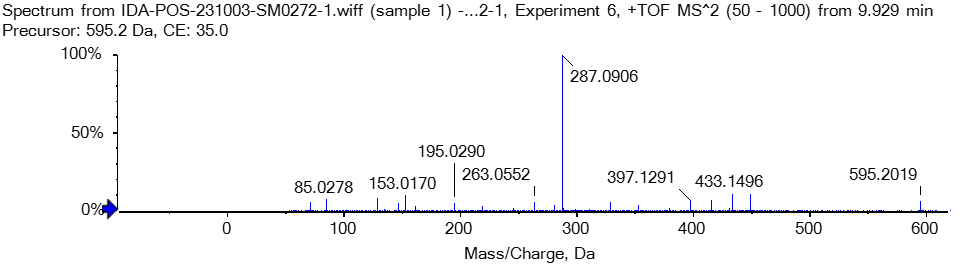** |
| **Fig.3. XIC chromatogram and MS spectra of Isosakuranetin 7*-O-*neohesperidoside (positive ionization mode)** |

|    |
| --- |
|  |
|  |
| **Fig. 4. XIC chromatogram and MS spectra of hesperetin (negative ionization mode)** |
|   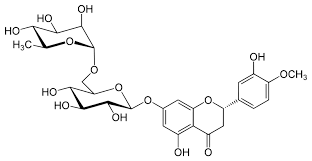 |
|  |
|  |
| **Fig. 5. XIC chromatogram and MS spectra of hesperidin (negative ionization mode)** |

|   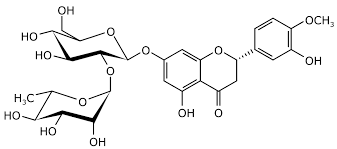 |
| --- |
|  |
|  |
| **Fig. 6. XIC chromatogram and MS spectra of hesperetin 7*-O-*neohesperidoside (negative ionization mode)** |

|    |
| --- |
|  |
|  |
| **Fig.7. XIC chromatogram and MS spectra of Eriodictyol 7*-O-*neohesperidoside (negative ionization mode)** |

| 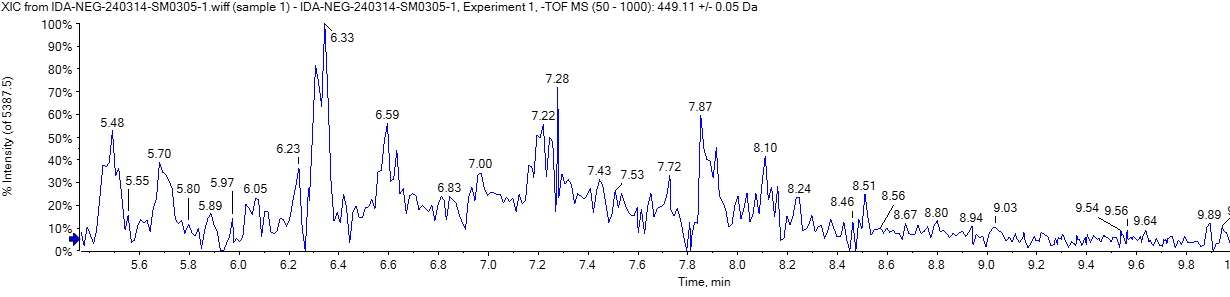   |
| --- |
|  |
|  |
| **Fig.8. XIC chromatogram and MS spectra of isookanin7*-O-*hexoside (negative ionization mode)** |

|   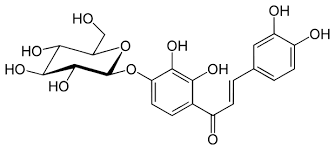 |
| --- |
|  |
| 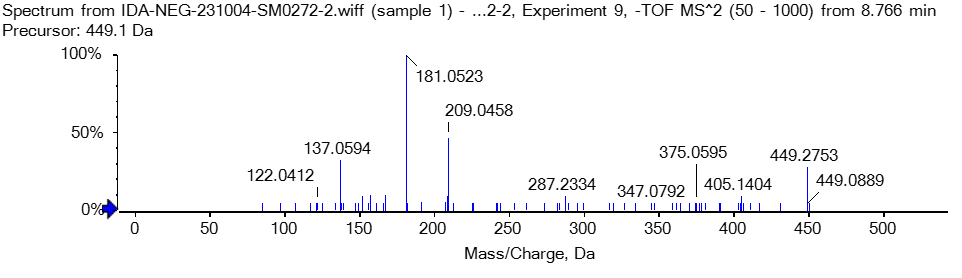 |
| **Fig.9. XIC chromatogram and MS spectra of okanin 4'*-O-*hexoside (negative ionization mode)** |

|   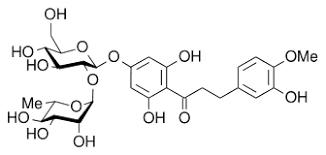 |
| --- |
|  |
|  |
| **Fig.10. XIC chromatogram and MS spectra of neohesperidin dihydrochalcone (negative ionization mode)** |

| 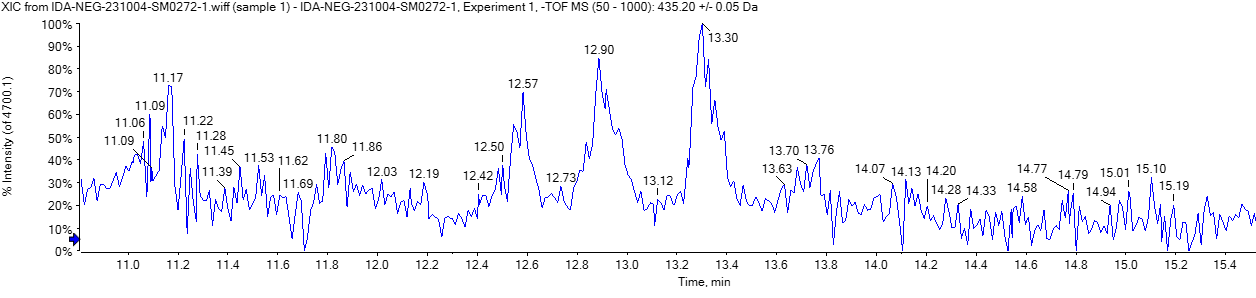  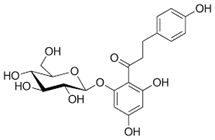 |
| --- |
| 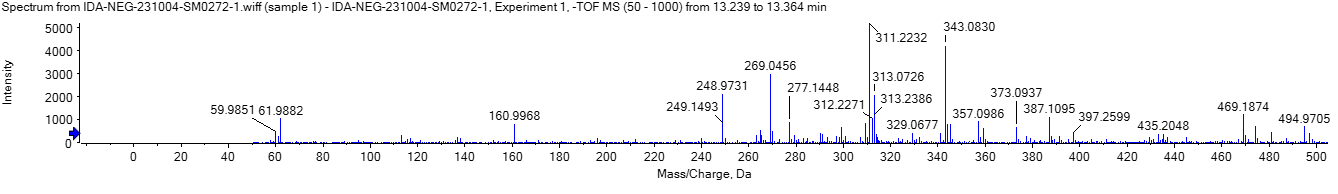 |
| 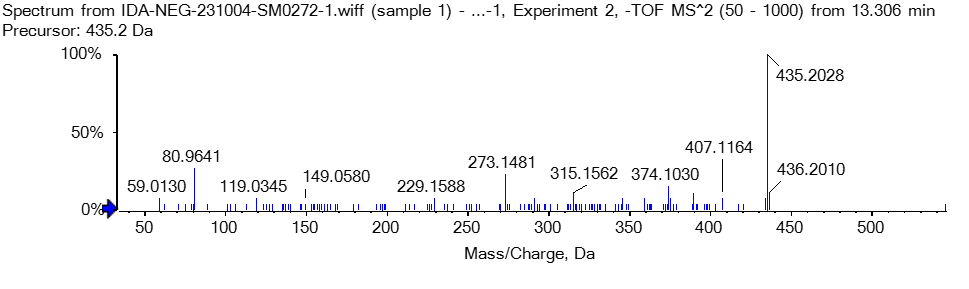 |
| **Fig.11. XIC chromatogram and MS spectra of Phlorizin (negative ionization mode)** |

|   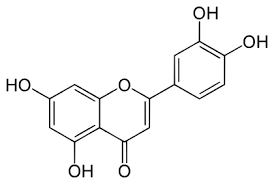 |
| --- |
|  |
|  |
| **Fig.12. XIC chromatogram and MS spectra of luteolin (negative ionization mode)** |
|    |
|  |
|  |
| **Fig.13. XIC chromatogram and MS spectra of luteolin 7,3'di*-O-*hexoside (negative ionization mode)** |

|   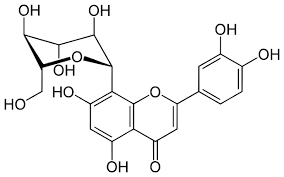 |
| --- |
|  |
|  |
| **Fig.14. XIC chromatogram and MS spectra of orientin (negative ionization mode)** |

|   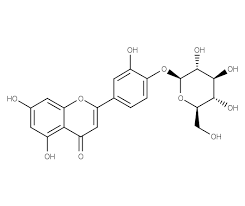 |
| --- |
|  |
|  |
| **Fig.15. XIC chromatogram and MS spectra of luteolin 4ꞌ*-O-*hexoside (positive ionization mode)** |

|    |
| --- |
|  |
|  |
| **Fig. 16. XIC chromatogram and MS spectra of Apigenin 8*-C-*hexoside (negative ionization mode)** |

|   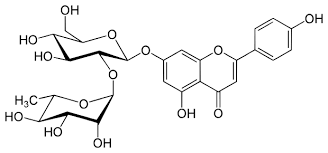 |
| --- |
|  |
|  |
| **Fig. 17. XIC chromatogram and MS spectra of Apigenin 7*-O-*neohesperidoside (Rhoifolin) (negative ionization mode)** |

|   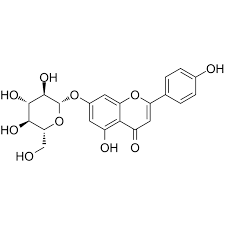 |
| --- |
|  |
|  |
| **Fig. 18. XIC chromatogram and MS spectra of apigenin 7*-O-*hexoside (negative ionization mode)** |

|   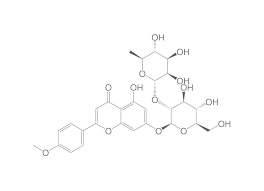 |
| --- |
|  |
|  |
| **Fig. 19. XIC chromatogram and MS spectra of acacetin 7*-O-*neohesperidoside (negative ionization mode)** |

|    |
| --- |
|  |
|  |
| **Fig. 20. XIC chromatogram and MS spectra of Apigenin (negative ionization mode)** |

|    |
| --- |
|  |
|  |
| **Fig. 21. XIC chromatogram and MS spectra of Acacetin (negative ionization mode)** |

|   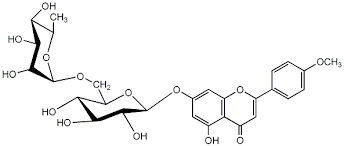 |
| --- |
|  |
|  |
| **Fig. 22. XIC chromatogram and MS spectra of Acacetin 7*-O-*rutinoside (negative ionization mode)** |

|   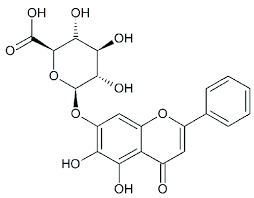 |
| --- |
|  |
|  |
| **Fig. 23. XIC chromatogram and MS spectra of baicalein 7*-O-*hexuronide (negative ionization mode)** |

|   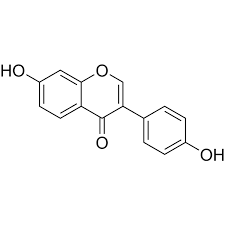 |
| --- |
|  |
|  |
| **Fig. 24. XIC chromatogram and MS spectra of daidzein (positive ionization mode)** |

|   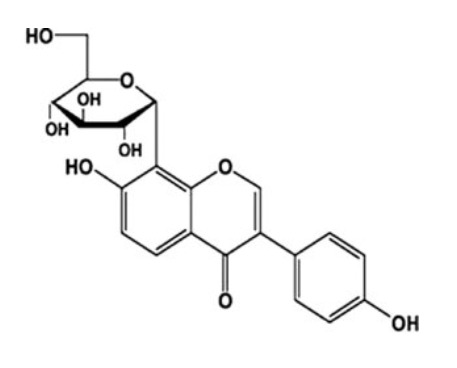 |
| --- |
|  |
|  |
| **Fig. 25. XIC chromatogram and MS spectra of daidzein 8*-C-*hexoside (puerarin) (negative ionization mode)** |

|   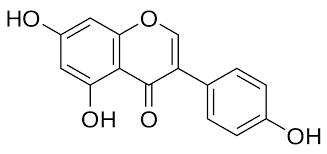 |
| --- |
|  |
|  |
| **Fig. 26. XIC chromatogram and MS spectra of genistein (positive ionization mode)** |

|   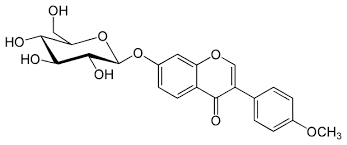 |
| --- |
|  |
|  |
| **Fig. 27. XIC chromatogram and MS spectra of ononin (positive ionization mode)** |

|   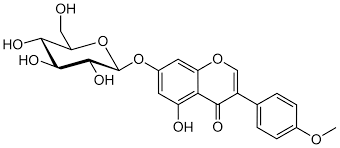 |
| --- |
|  |
|  |
| **Fig. 28. XIC chromatogram and MS spectra of sissotrin (positive ionization mode)** |

|   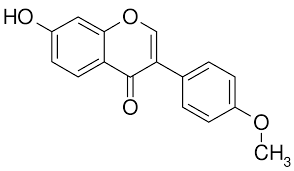 |
| --- |
|  |
|  |
| **Fig. 29. XIC chromatogram and MS spectra of formononetin (negative ionization mode)** |

|   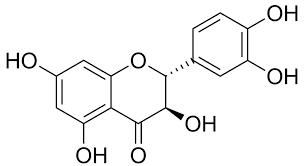 |
| --- |
|  |
|  |
| **Fig. 30. XIC chromatogram and MS spectra of taxifolin (positive ionization mode)** |

|   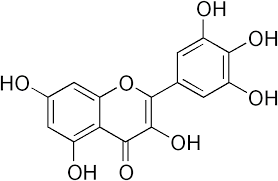 |
| --- |
|  |
|  |
| **Fig. 31. XIC chromatogram and MS spectra of myricetin (negative ionization mode)** |

|   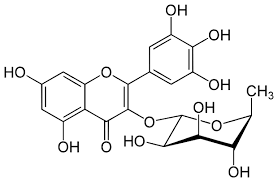 |
| --- |
|  |
|  |
| **Fig. 32. XIC chromatogram and MS spectra of myricetin 3*-O-*deoxyhexoside (myricitrin) (negative ionization mode)** |

|   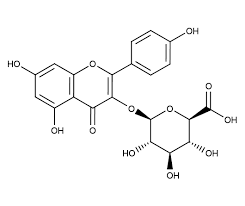 |
| --- |
|  |
|  |
| **Fig. 33. XIC chromatogram and MS spectra of kaempferol 3*-O-*hexuronide** **(negative ionization mode)** |

| 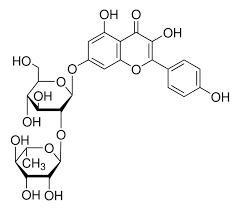 |
| --- |
|  |
|  |
| **Fig. 34. XIC chromatogram and MS spectra of kaempferol 7*-O-*neohesperidoside** **(negative ionization mode)** |

|  |
| --- |
|  |
|  |
| **Fig. 35. XIC chromatogram and MS spectra of kaempferol 3*-O-*robinoside-7*-O-*deoxyhexoside (positive ionization mode)** |

|  |
| --- |
|  |
|  |
| **Fig. 36. XIC chromatogram and MS spectra of kaempferol 3*-O-* α -L-deoxyhexoside (negative ionization mode)** |

| 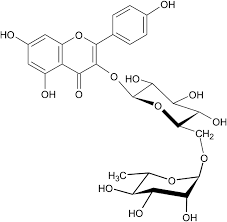 |
| --- |
|  |
|  |
| **Fig. 37. XIC chromatogram and MS spectra of kaempferol 3*-O-*rutinoside (positive ionization mode)** |

| 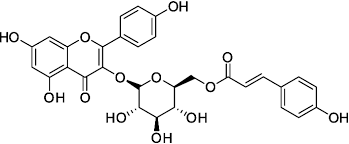 |
| --- |
|  |
|  |
| **Fig. 38. XIC chromatogram and MS spectra of kaempferol 3*-O-*(6-*p*-coumaroyl)-hexoside (negative ionization mode)** |

| 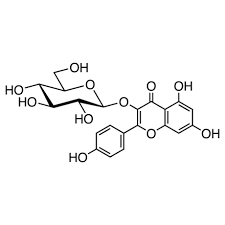 |
| --- |
|  |
|  |
| **Fig. 39. XIC chromatogram and MS spectra of kaempferol 3*-O-*hexoside (positive ionization mode)** |

|  |
| --- |
|  |
|  |
| **Fig. 40. XIC chromatogram and MS spectra of 3,5,7-trihydroxy-4'-methoxyflavone (kaempferide) (negative ionization mode)** |

| 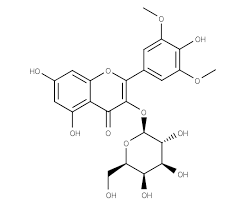 |
| --- |
|  |
|  |
| **Fig. 41. XIC chromatogram and MS spectra of syringetin 3*-O-*hexoside** **(negative ionization mode)** |

| 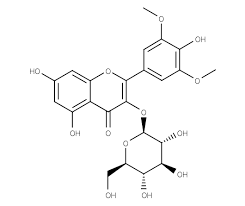 |
| --- |
|  |
|  |
| **Fig. 42. XIC chromatogram and MS spectra of syringetin 3*-O-*hexoside (negative ionization mode)** |

| 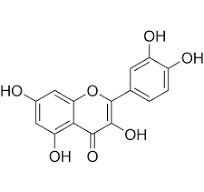 |
| --- |
|  |
|  |
| **Fig. 43. XIC chromatogram and MS spectra of quercetin (negative ionization mode)** |

| 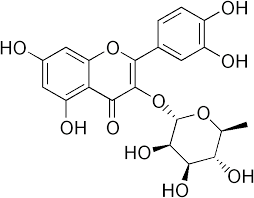 |
| --- |
|  |
|  |
| **Fig. 44. XIC chromatogram and MS spectra of quercetin 3*-O-*α-L-deoxyhexoside (quercitrin) (negative ionization mode)** |

| 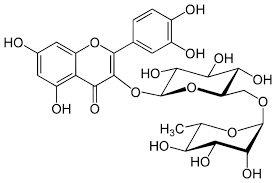 |
| --- |
|  |
|  |
| **Fig. 45. XIC chromatogram and MS spectra of quercetin 3*-O-*rutinoside (rutin) (positive ionization mode)** |

| 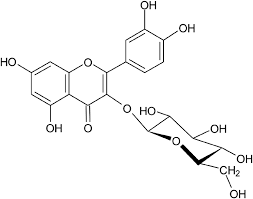 |
| --- |
|  |
|  |
| **Fig. 46 (a). XIC chromatogram and MS spectra of quercetin 3*-O-*hexoside (isoquercitrin) (positive ionization mode)** |

|  |
| --- |
|  |
|  |
| **Fig. 46 (b). XIC chromatogram and MS spectra of quercetin 4'*-O-*hexoside (positive ionization mode)** |

|  |
| --- |
|  |
|  |
| **Fig. 55. XIC chromatogram and MS spectra of quercetin 3*-O-*pentoside (negative ionization mode)** |

| 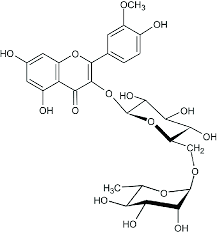 |
| --- |
|  |
|  |
| **Fig. 47. XIC chromatogram and MS spectra of isorhamnetin 3*-O-*rutinoside** **(negative ionization mode)** |

| 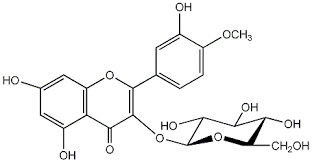 |
| --- |
|  |
|  |
| **Fig. 48. XIC chromatogram and MS spectra of isorhamnetin 3*-O-*hexoside** **(negative ionization mode)** |

| 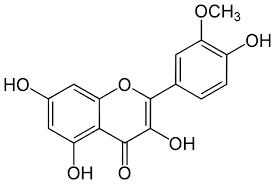 |
| --- |
|  |
|  |
| **Fig. 49. XIC chromatogram and MS spectra of 5,7,4'-trihydroxy-3'-methoxyflavonol aglycone (isorhamnetin) (negative ionization mode)** |

|  |
| --- |
|  |
|  |
| **Fig. 50. XIC chromatogram and MS spectra of delphinidin 3*-O-*(6ꞌꞌ*-O-*α-deoxyhexopyranosyl-β-hexopyranoside) (negative ionization mode)** |

| 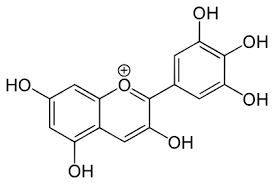 |
| --- |
|  |
|  |
| **Fig. 51. XIC chromatogram and MS spectra of delphinidin (negative ionization mode)** |

|  |
| --- |
|  |
|  |
| **Fig. 52. XIC chromatogram and MS spectra of malvidin 3*-O-*hexoside (negative ionization mode)** |

| 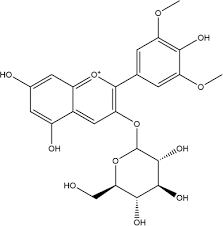 |
| --- |
|  |
|  |
| **Fig. 53. XIC chromatogram and MS spectra malvidin 3*-O-*hexoside (negative ionization mode)** |

| 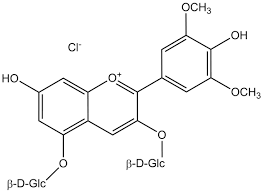 |
| --- |
|  |
|  |
| **Fig. 54. XIC chromatogram and MS spectra of malvidin 3,5-di*-O-*hexoside (positive ionization mode)** |

| 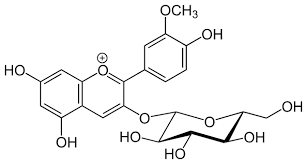 |
| --- |
|  |
|  |
| **Fig. 55. XIC chromatogram and MS spectra of peonidin 3*-O-*hexoside (negative ionization mode)** |

| 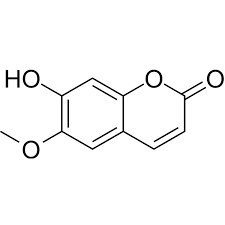 |
| --- |
|  |
|  |
| **Fig. 56. XIC chromatogram and MS spectra of scopoletin (negative ionization mode)** |

| 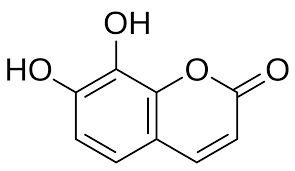 |
| --- |
|  |
|  |
| **Fig. 57. XIC chromatogram and MS spectra of daphnetin (negative ionization mode)** |

| 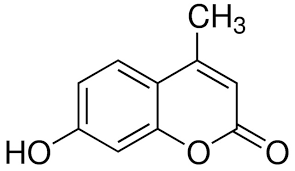 |
| --- |
|  |
|  |
| **Fig. 58. XIC chromatogram and MS spectra of 7-hydroxy-4-methyl-coumarin (negative ionization mode)** |

| 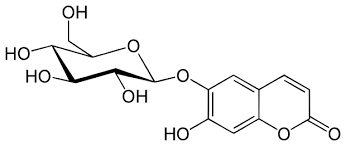 |
| --- |
|  |
|  |
| **Fig. 59. XIC chromatogram and MS spectra of esculin (negative ionization mode)** |

| 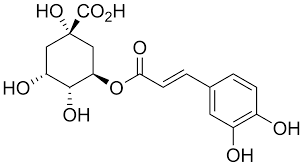 |
| --- |
|  |
|  |
| **Fig. 60. XIC chromatogram and MS spectra of chlorogenic acid (negative ionization mode)** |

|  |
| --- |
|  |
|  |
| **Fig. 61. XIC chromatogram and MS spectra of 1*-O-β*-D-hexopyranosyl sinapate**  **(negative ionization mode)** |

| 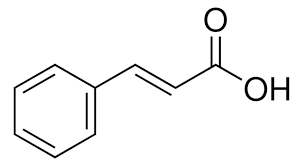 |
| --- |
|  |
|  |
| **Fig. 62. XIC chromatogram and MS spectra of *trans-*cinnamate (negative ionization mode)** |

| 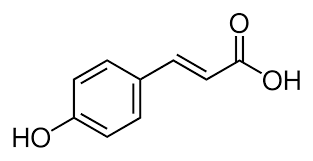 |
| --- |
|  |
|  |
| **Fig. 63. XIC chromatogram and MS spectra of *p-*coumaric acid (3-(4-hydroxyphenyl)-prop-2-enoic acid) (positive ionization mode)** |

| 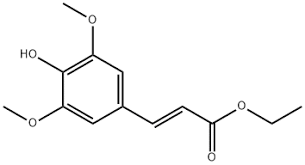 |
| --- |
|  |
|  |
| **Fig. 64. XIC chromatogram and MS spectra of 3-(4-hydroxy-3,5-dimethoxyphenyl)-2-propenoic acid (Sinapic acid) (positive ionization mode)** |

| 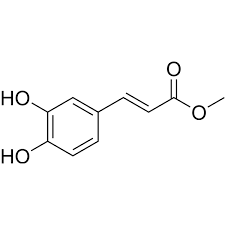 |
| --- |
|  |
|  |
| **Fig. 65. XIC chromatogram and MS spectra of caffeic acid (negative ionization mode)** |

| 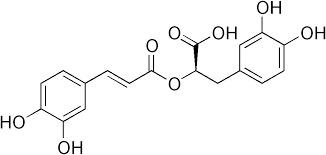 |
| --- |
|  |
|  |
| **Fig. 66. XIC chromatogram and MS spectra of rosmarinic acid (negative ionization mode)** |
